# Monophthalates of betulinic acid and related pentacyclic triterpenes inhibit efficiently the SOS-mediated nucleotide exchange and impact PI3K/AKT signaling in oncogenic K-RAS4B proteins†

**DOI:** 10.1039/d4ra08503e

**Published:** 2025-01-10

**Authors:** Gerrit E. Benary, Frank Kilgenstein, Sascha Koller, Jürgen Scherkenbeck

**Affiliations:** a University of Wuppertal, School of Mathematics and Natural Sciences Gaussstrasse 2042119 Wuppertal Germany scherkenbeck@uni-wuppertal.de

## Abstract

Betulinic acid and other herbal pentacyclic triterpenes have attracted interest in cancer research as these natural products induce apoptosis and suppress tumor progression. However, the molecular basis of the antitumor effect is still unknown. Here we show that monophthalates of betulinic acid and related triterpenes inhibit GDP/GTP exchange in oncogenic K-RAS4B proteins *via* the PI3K/AKT downstream cascade. According to a binding model based on molecular modelling, these derivatives act like a molecular glue that stabilizes an unproductive K-RAS4B^allo^:SOS complex. This represents a new mode of action and could be an attractive route for targeting RAS-related cancers.

## Abstract 2

36436308255New derivatives of betulinic, ursolic, and oleanolic acid inhibit GDP/GTP exchange at low micromolar levels and act as a molecular glue that stabilizes an unproductive K-RAS4B^allo^:SOS complex.

## Graphical abstract

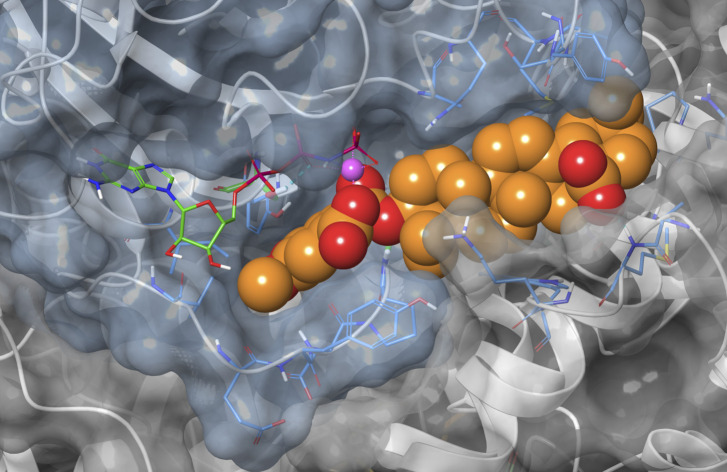

## Introduction

**Fig. 1:**
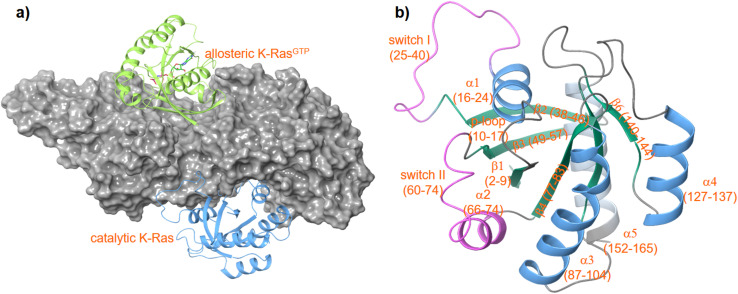
Structure of a K-RAS4B^cat^:SOS:K-RAS4B^allo^ complex (a) PDF code: 7KFZ and structure of K-RAS4B (b) PDB code: 4OBE.

K-RAS is a small GTPase belonging to the RAS superfamily of guanine nucleotide-binding proteins. RAS proteins interact *via* the well-established RAS-RAF-MEK-ERK and RAS-PI3K-PDK1-AKT signaling cascades that govern several crucial cellular processes such as cell growth, cell regulation, and cell proliferation. Mutations of the RAS isoforms K-RAS, H-RAS and N-RAS play a decisive role in lung, colorectal, and pancreatic cancer, the most common and life-threatening cancers overall. The incidence of a K-RAS4B mutation in colorectal cancer is around 50%, in pancreatic tumors even up to 90%.^1^ Thus, K-RAS4B has attracted widespread attention in cancer drug development. As a central switch K-RAS4B is highly regulated and toggles between an active GTP-bound and an inactive GDP-bound state. The nucleotide exchange in RAS-proteins is regulated by a specific guanine exchange factor (GEF) and a GTPase activation protein (GAP). The GEF ‘Son of Sevenless’ (SOS) catalyses the release of GDP and thus acts as an activator, while GAP increases the intrinsic RAS-GTPase activity and thereby inactivates RAS proteins.^2–4^ Inactive cytosolic SOS is recruited to the plasma membrane by binding first to a (GTP)RAS protein at an allosteric binding site located between the CDC25 and Rem domains of SOS.^5^ This process activates SOS to catalyze the release of GDP from a second RAS protein bound to the catalytic site of SOS (Fig. 1). The tight regulation of the GDP/GTP exchange is crucial for normal cell proliferation and survival.

**Fig. 2:**
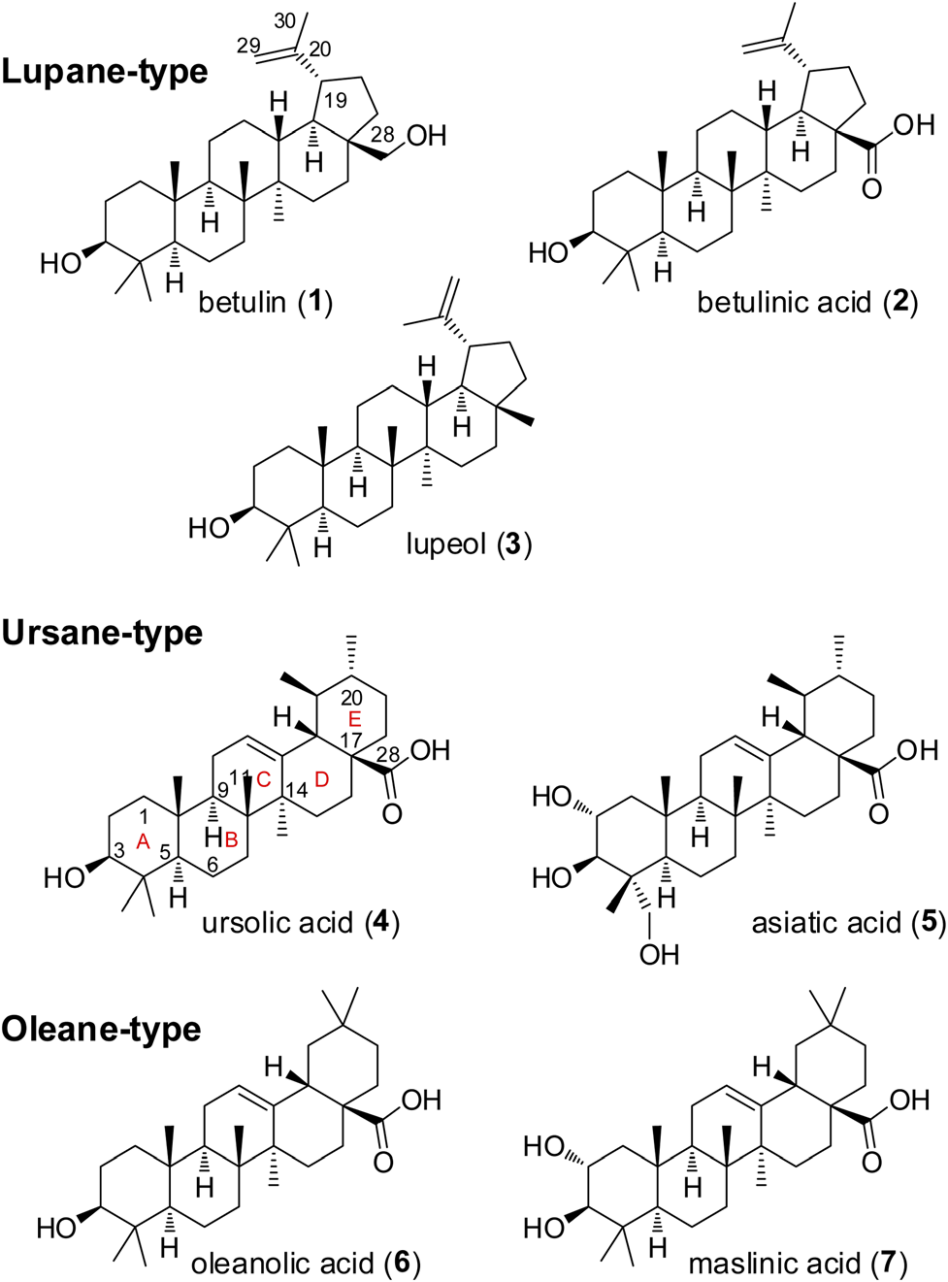
Structures of selected natural pentacyclic triterpenes.

Pentacyclic triterpenes constitute a large class of complex herbal natural products, that can be divided into the lupane, ursane, and oleane types (Fig. 2).^6,7^ Common plant families that are rich in these compounds include Asteraceae, Fabaceae, and Rosaceae.^8^ A notable example of a plant containing pentacyclic triterpenes is *Centella asiatica*, commonly known as gotu kola, which has been used in traditional Chinese medicine for its wound healing and anti-inflammatory properties.^9,10^ Several pentacyclic triterpenes (Fig. 2) have attracted attention in cancer research due to their cytotoxic effects.^11–13^ Mechanistic studies have demonstrated the ability of betulinic acid (2) to induce apoptosis, or programmed cell death, in a variety of cancer cell lines. A recent target identification-based analysis of cervical cancer cell apoptosis identified eight potential key targets for betulinic acid (2), including the long-known AKT.^14^ Other reported cellular effects of betulinic acid (2) encompass inhibition of angiogenesis and suppression of tumor progression as well as disruption of mitochondrial function leading to release of cytochrome c and activation of caspases.^15–17^ Ursolic acid (4) has been found to inhibit the proliferation of cancer cells by modulating multiple signaling pathways.^18,19^ Among others it suppresses the activation of NF-κB, a key regulator of inflammation and cancer.^20,21^ Furthermore, oleanolic acid (6) was shown to exert its antiproliferative effect on K-RAS-transformed cells by autophagy.^22^ In recent years, numerous derivatives of natural pentacyclic triterpenes have been prepared that exhibit improved or altered anticancer activities.^14,23–25^ Several literature reports indicate that in particular ursolic and betulinic acid derivatives also inhibit RAS dependent malignant cell-lines, in particular those with G12D and G13D mutations.^12,16,17,26–28^ In addition, an *in vivo* antitumor effect in K-RAS^G12D^ mice has been observed for SYK023, the *p*-methoxy-phenylacetic ester of betulinic acid.^29^ However, the molecular basis of the RAS-inhibitory effect is still unclear.

In the context of our ongoing search for new RAS inhibitors, we were interested in the more general question of whether pentacyclic triterpene acid derivatives could inhibit K-RAS4B by interfering with the guanine exchange process.

## Results and discussion

### Chemical syntheses

**Fig. 3:**
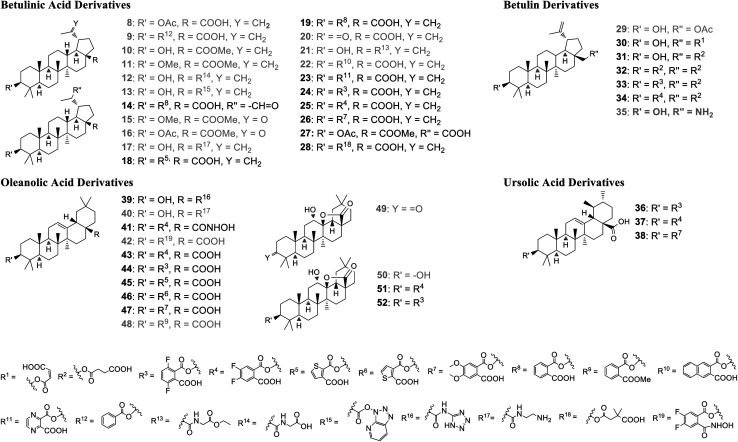
Pentacyclic triterpenes derivatives prepared for biological evaluation. Grey color: compounds regarded as inactive in the GDP/GTP exchange assay (IC_50_ > 100 μM).

All compounds (Fig. 3) described here, were prepared from the natural triterpenes betulin (B), betulinic acid (BA), oleanolic acid (OA), and ursolic acid (UA) according to literature procedures. In order to establish the significance of the 3-hydroxy group for GDP/GTP exchange inhibition, BA was acetylated and benzoylated to afford derivatives 8 (55%) and 9 (42%), respectively.^30,31^ The phthalic acid monoesters of all pentacyclic triterpenes were prepared in varying yields according to Kvasnika's method described for unsubstituted phthalic anhydride.^32^ Methylation of BA with MeI gave ester 10 in 60% and the dialkylation product 11 in 32% yield.^33^ The glycinamide derivative 12 was obtained by HATU-mediated coupling of ethyl glycinate (R = R^13^)and subsequent hydrolysis of the ester in a yield of 63% over both steps.^34^ Due to the steric hindrance and associated lower reactivity of the axial carboxy group, the unreacted hydroxy-azabenzotriazole intermediate 13 could also be isolated in low yield (7%).^35^ The C-20 external double bond of compound 19 was converted with *m*CPBA into the corresponding epoxide, which rearranged instantaneously to aldehyde 14. The double bond of protected BA could be oxidized to the ketones 15 and 16 either by ozonolysis (37%) or by a Lemieux–Johnson oxidation with NaIO_4_/RuO_4_ (44%).^36,37^

Betulin contains an equatorial secondary hydroxy group at position 3 and an axial hydroxymethyl group at position 28, whose reactivity is further reduced by the neopentyl environment. Nevertheless, the primary OH-group is still the more reactive one and can be selectively mono-acetylated to derivative 29.^38^

A similar chemoselectivity was observed with maleic and succinic anhydride in the preparation of compounds 30 and 31.^24,39^ Only a small amount (10%) of the double succinylated product 32 was isolated.^40^

In addition to the BA derivatives, the C-17 carboxy group of OA was converted into the bioisosteric tetrazole derivative 39 (23%).^41^ The reaction of OA with *N*-Boc-ethylendiamine and subsequent deprotection with dilute HCl yielded the ethylamine derivative 40 (78%).^42^ In order to further evaluate the role of the carboxy group in position 17, hydroxamic acid derivative 41 was prepared from oleanolic acid *via* a four step sequence, including acetylation of the 3-hydroxy group, hydroxamic acid formation with hydroxylamine, deprotection of the 3-acetyl group, followed by reaction with 4,5-difluorophthalic anhydride.

The oleanoyl 4,5-difluoro-2-(hydroxycarbamoyl)benzo-ate 42 was obtained in low yield by direct conversion of 4,5-difluorophthalic monoester 43 with hydroxylamine. The ketolactone 49 was obtained in one-step by ozonolysis of OA in 77% yield. Subsequent stereoselective reduction with NaBH_4_ afforded in 92% yield the hydroxylactone 50 which was finally converted into the 3-monoester derivatives 51 and 52.^43,44^

### Biological evaluation and mode of action studies

**Fig. 4:**
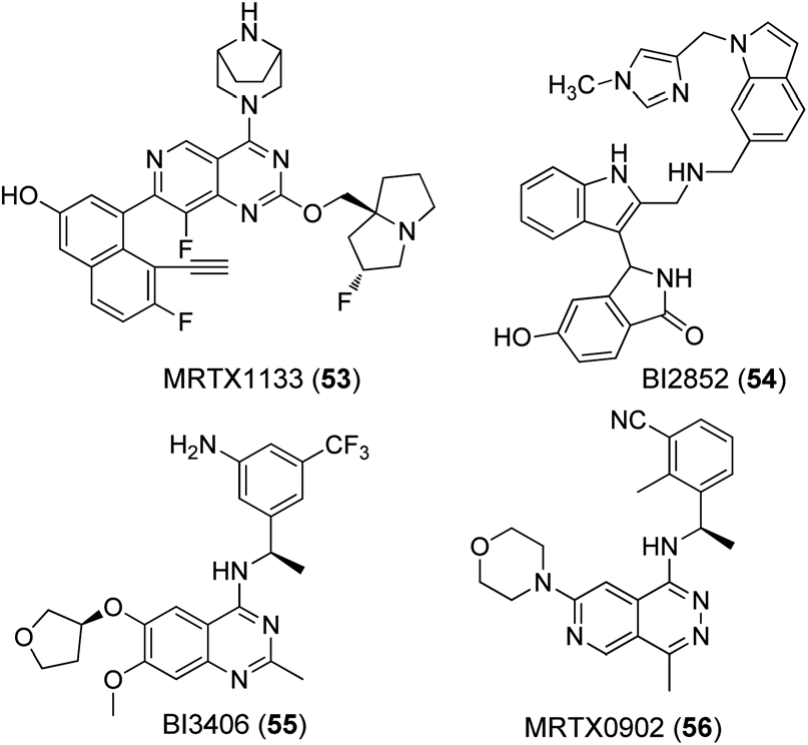
Known ligands for K-RAS4B:SOS interaction sites.

A nucleotide exchange assay was used as the primary test system for all compounds (Table 1). This assay measures the SOS mediated release of GDP by conversion to ATP, which is quantified by a luciferase/luciferin reaction.^45^ The well-known K-RAS4B inhibitors MRTX1133 (53) and BI2852 (54) as well as the SOS-inhibitor BI3406 (55) were tested as reference compounds (Fig. 4). MRTX1133 (53) is a clinical development candidate which specifically binds to the SII-site of K-RAS4B and inhibits the K-RAS4B:SOS-interaction at a low nanomolar level while BI2852 (54) represents a ligand for the SI/SII-site and inhibits the K-RAS4B:SOS-interaction at the micromolar level, which is a characteristic value for ligands of the SI/SII site.^42,46,47^ The same effect is achieved with BI3406 (55) and MRTX0902 (56), which bind to SOS and also prevent the formation of the K-RAS4B:SOS complex and subsequent GDP/GTP exchange.^48,49^ Consequently, the K-RAS protein remains in the GDP-bound, inactive state.

**Table 1:** Inhibition of K-RAS4B GDP/GTP exchange by pentacyclic triterpene derivatives^a^

Cmpd	Wild-type IC_50_ [μM]	G12D IC_50_ [μM]	G12V IC_50_ [μM]	Solubility [μM]
**Derivatives of betulinic acid**				
14	37.3 ± 19.1	48.5 ± 12.7	51.1 ± 6.2	447
18	1.27 ± 0.69	1.83 ± 1.45	n.d.	255
19	15.3 ± 3.8	10.4 ± 4.7	16.6 ± 5.2	191
23	23.3 ± 2.9	11.8 ± 1.0	9.61 ± 4.82	434
24	15.7 ± 4.3	8.63 ± 3.72	15.4 ± 2.9	378
25	39.1 ± 5.4	5.00 ± 0.91	9.79 ± 3.41	240
26	>100	36.1 ± 14.7	26.4 ± 3.9	158
27	n.d.	13.8 ± 4.2	n.d.	n.d.
28	2.15 ± 0.17	0.59 ± 0.19	0.61 ± 0.14	n.d.

**Derivatives of betulin**				
30	69.2 ± 6.8	6.80 ± 2.32	3.19 ± 0.19	n.d.
31	25.6 ± 4.3	10.4 ± 0.8	12.7 ± 5.5	n.d.
32	1.01 ± 0.31	0.71 ± 0.18	1.37 ± 0.28	n.d.
33	2.09 ± 0.59	1.45 ± 0.93	2.13 ± 0.87	416
34	1.18 ± 0.02	0.66 ± 0.13	2.85 ± 0.14	410

**Derivatives of ursolic acid**				
36	3.46 ± 1.94	2.64 ± 1.75	2.52 ± 0.99	424
37	3.94 ± 1.02	2.53 ± 0.63	2.15 ± 0.31	220
38	53.6 ± 7.3	55.6 ± 6.5	n.d.	383

**Derivatives of oleanolic acid**				
39	12.8 ± 2.9	27.8 ± 2.6	9.18 ± 0.99	n.d.
41	4.68 (*)	7.61 ± 0.70	5.47 (*)	n.d.
43	1.50 ± 0.08	2.22 ± 0.29	2.71 ± 0.39	394
44	1.33 ± 0.24	2.88 ± 0.89	2.51 ± 0.24	424
45	1.52 ± 0.50	3.06 ± 0.79	5.62 ± 0.93	390
46	4.15 ± 1.32	3.67 ± 0.35	8.12 ± 1.24	421
47	2.71 ± 1.30	1.61 ± 0.85	2.90 ± 0.51	302
51	7.18 ± 0.68	26.5 ± 5.9	8.57 ± 1.09	99
52	10.6 ± 2.9	27.1 ± 4.2	8.70 ± 0.59	320

**Reference compounds**				
53	<0.3	<0.03	<0.03	322
54	13.4 ± 2.1	13.7 ± 5.1	10.9 ± 0.6	114
55	0.13 ± 0.08	<0.03	<0.03	277

a Values were calculated from eight data points. At least three independent determinations were performed. The asterisk (*) indicates a single determination. n.d.: not determined.

Disappointingly, all tested natural pentacyclic triterpenes 1–7 (Fig. 2) did not show any notable inhibition of GDP/GTP exchange for the most frequent K-RAS4B^G12D^ mutant. In particular, for betulinic and ursolic acid IC_50_ values of >300 μM were measured. However, a significant inhibition of GDP/GTP exchange was found for the semi-synthetic betulinic monophthalic ester 19 (IC_50_ 10.4 ± 4.7 μM) which has already been described as a cytotoxic compound with IC_50_ values in the range of up to 10 μM against a series of cancer cell lines.^29^

To establish an initial structure activity model, we prepared several betulinic acid derivatives, all of which were tested for their inhibition of the G12D and the more challenging G12V mutant, as well as their selectivity for K-RAS4B^wt^ (Table 1). Acylation of the 3-OH group (8, 9) and formation of esters or amides of the 17-carboxy group (10, 21) led to inactive betulinic acid derivatives. The inactivity of these lipophilic derivatives cannot be a mere solubility effect, as shown by the glycine-derived acid 12, the polar ester 13, and the amine 17, all of which were inactive, although their solubility is comparable to that of the reference compound BI2852 (53).

The relevance of the 3-OH group is further corroborated by betulonic acid 20, the 3-keto derivative of BA, which was also inactive. The platanic acid derivatives 15 and 16, obtained by oxidation of the 19-isopropenyl group of betulinic acid, had a limited solubility and IC_50_ values could be measured only up to 30 μM. The well soluble monophthalic acid ester 14 (IC_50_ 48.5 ± 12.7 μM) showed lower inhibition of the GDP/GTP exchange than the parent compound 19 (IC_50_ 10.4 ± 4.7 μM), suggesting that an aldehyde function is not a suitable substitute for the isopropenyl group. On the other hand, oxidation of the isopropenyl group led to the corresponding carboxylic acid 27, which showed GDP/GTP exchange inhibition (13.8 ± 4.2 μM) at the same level as BA derivative 19, even with the fully protected 3-hydroxy and 17-carboxy group. Taking these initial data together, it becomes evident that the SAR of betulinic acid is quite narrow.

In a second step, we therefore focused on phthalic mono-esters. Unfortunately, even the change from phthalic (19) to 2,3-naphthoic monoester 22 led to a loss of GDP/GTP exchange inhibition. The electron-rich 4,5-dimethoxy phthalate 26 exhibited only moderate activity in the G12D mutant. On the other hand, the replacement of the phenyl ring by an electron-deficient pyrazine-2,3-dicarboxylic monoester (23) or the introduction of electron-withdrawing fluorine atoms (24) led to betulinic acid derivatives with similar activity as the parent compound 19. Both betulinic acid derivatives had similar activities for the G12D and G12V mutant.

The corresponding 4,5-difluorophthalic monoester 25 showed a somewhat improved inhibition (IC_50_ 5.00 ± 0.91 μM) of GDP/GTP exchange for K-RAS4B^G12D^ and a slightly lower activity for the G12V mutant but a significant selectivity by a factor of 4–6 for K-RAS4B^wt^. The thiophene derivative 18 has an impressive IC_50_ value (G12D mutant) of 1.83 ± 1.45 μM, which corresponds to an improvement of at least a factor of five compared to compound 19, but completely at the expense of selectivity for K-RAS4B^wt^. Most strikingly, the HIV inhibitor bevirimat (28) inhibited the GDP/GTP exchange at high nanomolar levels for both the G12D (IC_50_ 0.59 ± 0.19 μM) and G12V (IC_50_ 0.61 ± 0.14 μM) mutant, with a selectivity of 3–4 fold (IC_50_ (wt) 2.15 ± 0.17 μM). This finding indicates, that betulinic acid derivatives modified in position 3 have the potential for nanomolar GDP/GTP exchange inhibitors. This is especially important for the G12V mutant, which is notoriously difficult to address due to the non-functionalized valine side-chain.

Those results encouraged us to extend our studies to related pentacyclic triterpenes such as betulin, oleanolic acid, and ursolic acid. In general, the structure activity relationships (SAR) were similar to betulinic acid (Fig. 3 and Table 1). Blocking of the hydroxy- and carboxy groups by esterification or by introduction of an amine function (35, 40) always resulted in inactive derivatives. Within the betulin series GDP/GTP exchange inhibition could be brought to a level equal to the best betulinic acid derivatives. In particular, betulin derivative 30 exhibits a good activity for the G12V mutant (IC_50_ 3.19 ± 0.19 μM) together with a remarkable selectivity by a factor of 15–20 (IC_50_ (wt) 69.2 ± 6.8 μM) for wildtype K-RAS4B. Introduction of a second succinic monoester or a 4,5-difluorophthalic monoester at position 3 resulted in betulin derivatives 32 and 34, both of which inhibited the GDP/GTP exchange in K-RAS4B^G12D^ in the high nanomolar range (0.71 ± 0.18 μM and 0.66 ± 0.13 μM), unfortunately again at the expense of selectivity. Dihydroxylation of the oleanolic acid Δ^12^-double bond and subsequent reduction of the 3-keto group with NaBH_4_ resulted in the formation of the highly stable but inactive hydroxylactone 50, demonstrating again the indispensability of a free carboxylic acid in position 17. Expectedly, only a reduced biological activity was found for the 4,5-difluorophthalic acid monoester 51 (IC_50_ 26.5 ± 5.9 μM) prepared from the hydroxylactone 50. In the oleanolic and ursolic acid series, best inhibitory activities in the low micromolar range were obtained with the same monoesters in position 3 as for betulinic acid. The oleanolic acid derivatives 43, 44, and 47 showed a promising activity against the G12V mutant. Unfortunately, all oleanolic acid derivatives exhibited no selectivity in favor of the oncogenic K-RAS4B mutants. Worthwhile to mention, the oleanolic 4,5-dimethoxy-phthalic monoester 47 retained activity while the corresponding derivatives of betulinic (26) and ursolic acid (38) were found significantly less active. The relevance of the free phthalic carboxy group is further corroborated by hydroxamic acid 42 and diester 48 which both turned out completely inactive.

For selected betulinic acid derivatives quantitative ERK and AKT phosphorylation was measured in SNU-1 (K-RAS4B^G12D^) and A375 cells (K-RAS4B^wt^) to determine whether pentacyclic triterpenes act *via* the RAS-RAF-MEK-ERK or the RAS-PI3K-PDK1-AKT downstream cascade (Table 2). The betulinic acid derivatives tested, had no activity on the ERK pathway, but inhibited AKT phosphorylation with micromolar IC_50_ values in agreement with literature reports.^50,51^ All betulinic acid derivatives showed selectivity for the K-RAS4B^G12D^ mutant cell line SNU-1. No activity was detected in A375 cells carrying a BRAF but no K-RAS mutation. Our results demonstrate, that betulinic acid derivatives not only inhibit GDP/GTP exchange in a biochemical assay but disrupt specifically downstream signalling *via* the RAS-PI3K-PDK1-AKT pathway in RAS mutant cells.

**Table 2:** Inhibition (IC_50_ [μM]) of ERK and AKT phosphorylation by betulinic acid derivatives^a^

Cmpd	SNU-1 pERK	SNU-1 pAKT	A375 pERK	A375 pAKT
19	>300	19.0	>1000	>1000
23	>1000	61.7	>1000	>1000
24	134	13.0	>1000	>1000
25	>300	18.1	>1000	>1000
26	>300	83.7	>1000	>1000

a In general, two independent determinations were performed.

However, neither the GDP/GTP exchange assay nor the ERK and AKT phosphorylation data provide any further information on the exact mode of action of the pentacyclic triterpene derivatives. In principle, the inhibition of GDP/GTP exchange can be achieved either by binding of the ligands to K-RAS4B, to SOS or to a K-RAS4B:SOS complex. K-RAS4B and even more K-RAS4B:SOS complex inhibitors with low micromolar or nanomolar IC_50_ values are still considered highly interesting.

**Fig. 5:**
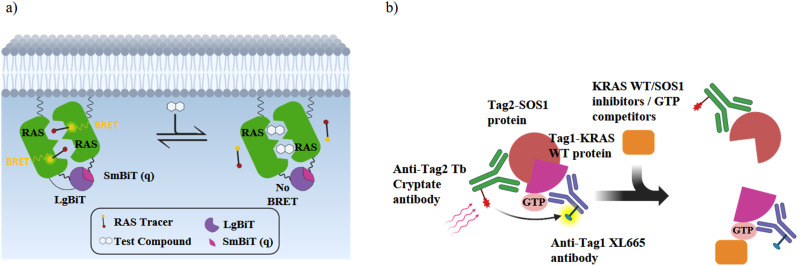
Schematic illustrations of the RAS cellular target engagement and RAS:SOS protein–protein interaction assay. (a) NanoBRET target engagement (TE) assay: when the fusion proteins LgBiT-RAS and SmBiT(q)-RAS interact in live-cells, they form an active luciferase as a BRET donor. The BRET receptor is a fluorescent NanoBRET® tracer that binds specifically to K-RAS. K-RAS binders displace the tracer and reduce the BRET signal in a dose-dependent manner. (b) K-RAS^wt^:SOS PPI assay: without an inhibitor, (GTP)K-RAS^wt^ binds to SOS and the binding of each detection reagent to its tagged target generates a HTRF signal. In the presence of K-RAS or SOS inhibitors or GTP competitors, the HTRF signal decreases in a dose-dependent manner.

To obtain further information on the mode of action, selected GDP/GTP exchange inhibitors were studied in additional assays (Table 3). A target engagement assay, which is based on bioluminescence resonance energy transfer (BRET), was used to measure target engagement at protein complexes in live cells (Fig. 5a). The BRET effect results from an energy transfer between the target luciferase fusion protein and a cell-permeable fluorescent tracer in intact cells. We used this assay to study the displacement of fluorescently labelled BI2852 (54) from its SI/SII-binding site.^52^ Expectedly, the SOS-binder BI3406 (55) did not show any effect. Noteworthy, the strong SII-site ligand MRTX1133 (53) also exhibited a nanomolar *K*_D_ value for the displacement of BI2852 (54) at the SI/SII-site. Apparently, binding of MRTX1133 (53) to the SII-site induces conformational shifts at the nearby SI/SII-site that release BI2852 (54).

**Table 3:** Further biological evaluation of selected pentacyclic triterpenes^a^

Cmpd	K-RAS4B^wt^:SOS PPI^b^*K*_D_ [μM]	K-RAS4B^wt^ NanoBRET TE^c^*K*_D_ [μM]	K-RAS4B^G12D^ NanoBRET TE^c^*K*_D_ [μM]
18	>50	21.8	20.1
32	>100	40.8	29.1
33	37.4	n.d.	26.4
34	>100	n.d.	>100
36	>50	n.d.	>50
37	>50	n.d.	16.6
47	>50	39.0	>50
53	0.09	0.068	0.042
55	0.014	n.d.	>100

a In general, two independent determinations were performed. n.d.: not determined.

b Protein–protein interaction assay of (GTP)K-RAS4B^wt^ and SOS.

c Target engagement cell-assay: displacement of BI2852 from the K-Ras4B SI/II binding-site.

Noteworthy, the majority of the particularly active GDP/GTP exchange inhibitors (32, 34, 36, 47) displace BI2852 (54) from the SI/SII-site only at higher concentrations. Since all these pentacyclic triterpene derivatives have a higher solubility than BI2852 (54), this is not a plausible explanation for the reduced activity in the NanoBRET assay. Rather, it seems more likely that the monophthalate derivatives bind to a different site.

The interaction of (GTP)K-RAS4B^wt^ and SOS was measured with a HTRF assay using an SOS-specific antibody labeled with terbium cryptate as HTRF (homogeneous time-resolved fluorescence) donor and a K-RAS4B^wt^ specific antibody labeled with an HTRF acceptor (XL665).^52^ Upon (GTP)K-RAS4B^wt^:SOS complex formation, excitation of the donor antibody triggers fluorescence resonance energy transfer (FRET) towards the acceptor antibody, which emits specifically at 665 nm. Therefore, K-RAS4B or SOS inhibitors, which cause dissociation of the K-RAS4B:SOS complex, reduce HTRF signaling (Fig. 5b). The K-RAS4B site-II ligand MRTX1133 (53) and especially the SOS ligand BI3406 (55) are potent inhibitors of K-RAS4B^wt^:SOS complex formation (Table 3).

Remarkably, a significant discrepancy in activity between the GDP/GTP exchange assay and the K-RAS4B^wt^:SOS interaction assay was observed for all pentacyclic triterpenes. The *K*_D_ values for dissociation of the K-RAS4B:SOS complex are a factor of 20–50 higher for all potent, single-digit micromolar GDP/GTP exchange inhibitors, indicating that GDP/GTP exchange is inhibited at a concentration where the K-RAS4B:SOS complex is still intact. Furthermore, surface plasmon resonance (SPR) measurements showed that the potent GDP/GTP exchange inhibitor bevirimat (28) does not bind to either K-RAS4B^G12D^ (*K*_D_ > 300 μM) or SOS (*K*_D_ > 1000 μM) alone.

These data imply that the pentacyclic triterpenes studied here do not act directly on K-RAS4B or SOS but potentially by interaction with the K-RAS4B:SOS complex. Such a mode of action would be very interesting to the field, as only a limited number of small molecules is known to interfere with the K-RAS4B:SOS complex.^53–57^ In order to develop a binding hypothesis, various molecular modeling studies were carried out with selected triterpene derivatives.

Prior to *in silico* docking with Glide (Schroedinger molecular modeling suite), a sufficient number of energetically favored conformers was generated by a conformational search (mixed torsional/low mode sampling) procedure. Unfortunately, all known interaction sites on K-RAS4B, SOS, and at the K-RAS4Bcat:SOS interface turned out to be too small for the pentacyclic triterpene scaffolds, leading to low docking scores (4–5 kcal mol^−1^) with questionable significance.^50,51^ As expected, insignificant docking scores were also found for K-RAS4B^cat^ in a K-RAS4B^allo^:SOS:K-RAS4B^cat^ complex (PDB code: 7KFZ).

**Fig. 6:**
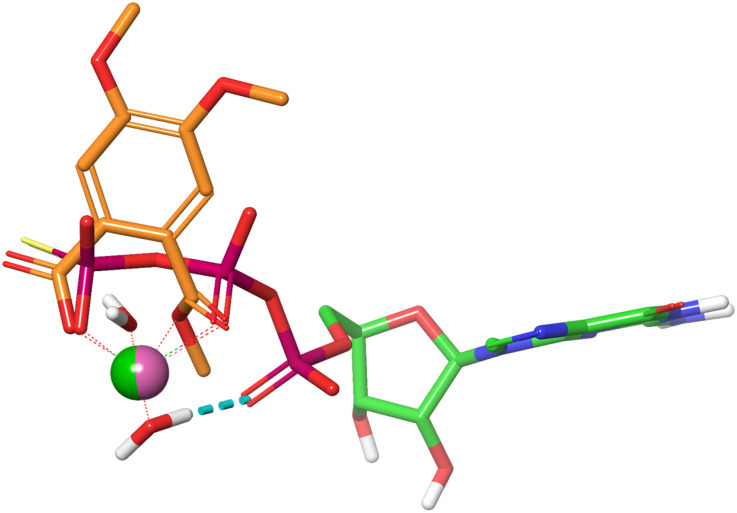
Superposition of GTPγS·Mg^2+^·2H_2_O (PDB code: 4DSO) and monomethyl Mg phthalate·2H_2_O.

Mg^2+^ is tightly bound to the triphosphate group of GTP through an ionic interaction with the γ-phosphate group, a coordinative bond to the neighboring β-phosphate, and a water mediated hydrogen bond to the α-phosphate residue. Together with a second water molecule Mg^2+^ forms an octahedral complex when bound to GTP (Fig. 6). An overlay of the energy-minimized dihydrate of magnesium mono (4,5-dimethoxy-2-(methoxycarbonyl)benzoate) and GTPγS·Mg^2+^·2H_2_O reveals an excellent match with both Mg^2+^ cations occupying almost identical positions. In this superposition the carboxy group of the monophthalate moiety agrees well with the γ-phosphate, and the ester carbonyl group with the phosphor oxygen double bond of the β-phosphate. Noteworthy, the quality of the overlay is considerably lower for the corresponding Mg^2+^·GDP·4H_2_O complex. The monophthalate building block, which is present in almost all active triterpene derivatives, can therefore be regarded as a Mg^2+^-chelator with a bite-angle similar to the terminal diphosphate in GTP. This finding prompted us to examine the monophthalate triterpenes for their interaction with the (Mg^2+^·GTP)K-RAS4B^allo^:SOS interface.

**Fig. 7:**
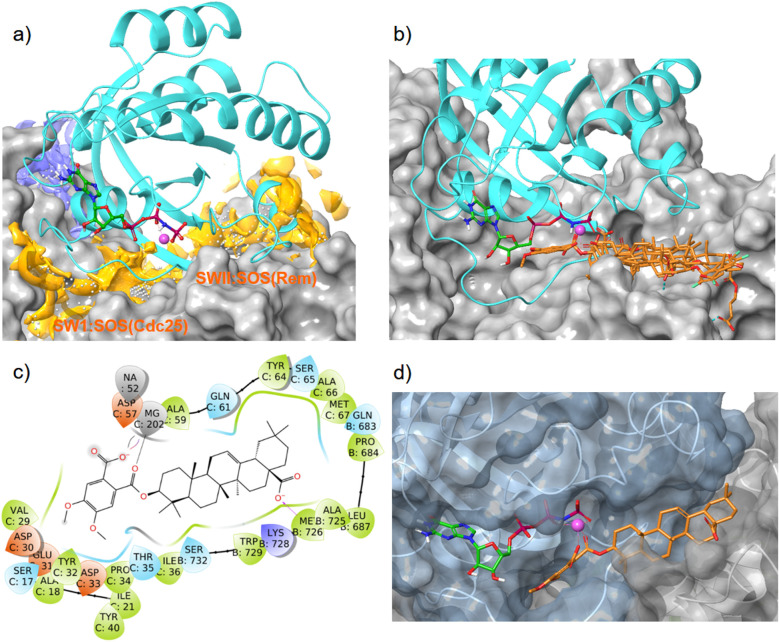
Interaction of oleanolic acid derivative 47 and related pentacyclic triterpene derivatives with the allosteric K-RAS4B:SOS interface (PDB code: 7KFZ). Cyan: K-RAS4B^allo^, magenta sphere: Mg^2+^, green carbons: GTP, orange carbons: pentacyclic triterpenes. (a) With SiteMap calculated binding sites (blue and orange). (b) Overlay of GDP/GTP exchange assay hits 34, 37, 38, 43, and 47 in the high-affinity SWII:SOS^Rem^ pocket. (c) Ligand protein interaction diagram of derivative 47 with the K-RAS4B:SOS^Rem^ domain; colors of the boxes indicate polarities of the amino acids. (d) MD simulation based lowest free binding energy (Δ*G* = −124 kcal mol^−1^) snapshot of compound 47 at the SWII:SOS^Rem^ interface.

A SiteMap search identified a smaller binding site (blue color) in the guanidine pocket region and a large major binding site (orange color), encompassing switch I, switch II, and the *α*_2_-helix of K-RAS4B^allo^ (Fig. 7a). While the small binding-site is too far away from the Mg^2+^ cation, the large site consists of two sub-pockets, both of which are able to accommodate a large pentacyclic triterpene scaffold and allow the formation of magnesium complexes.

*In silico* dockings at the K-RAS4B:SOS^Cdc25^ subpocket (Fig. 7a) revealed only low docking-scores due to steric clashes with the guanosine triphosphate in close proximity. In contrast, the docking scores for the K-RAS4B:SOS^Rem^ subpocket (Fig. 7a) were good to excellent (Table 4). In particular, compounds 38 and 47 showed docking-scores comparable to GDP (PDB code 4DSU), but were inferior to the stable GTP analogue GppNHP (PDB code 7KFZ), which is known to bind significantly stronger to K-RAS4B than GDP. All GDP/GTP exchange assay hits examined (Table 4) bind to the high-affinity K-RAS4B^allo^:SOS^Rem^ site in a similar manner (Fig. 7b).

**Table 4:** Docking scores and binding energies of triterpene derivatives to the K-RAS4B^allo^:SOS^Rem^ subpocket

Ligand	Protein (PDB code)	Docking score (kcal mol^−1^)	Δ*G* binding (kcal mol^−1^)
GDP	4DSU	−12.7	−73.6
GppNHP	7KFZ	−16.1	−78.8
34	7KFZ	−10.3	−88.1
37	7KFZ	−7.13	−76.4
38	7KFZ	−12.2	−102
43	7KFZ	−7.78	−70.4
47	7KFZ	−11.6	−106

To obtain additional information on the binding mode, a 500 ns molecular dynamics simulation was conducted for oleanolic acid derivative 47 bound to the high-affinity K-RAS4B^allo^:SOS^Rem^ site of the crystal structure 7KFZ. A trajectory with a total of 2000 frames was created by taking snapshots every 250 ps. MM/GBSA calculations were performed for a total of 1000 frames previously extracted from the trajectory. The mean Δ*G* binding energy was −60.2 ± 12.4 kcal mol^−1^ with a maximum value of −124 kcal mol^−1^.

**Fig. 8:**
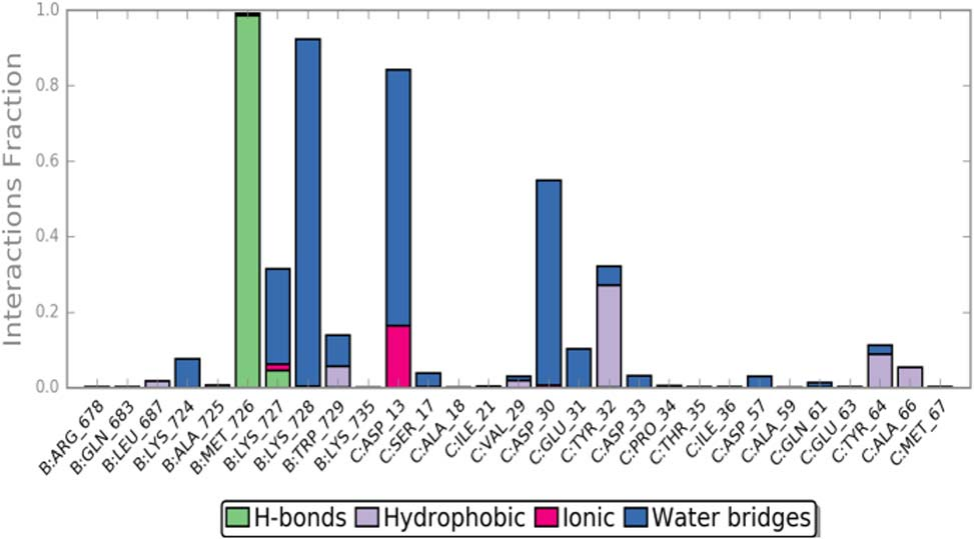
Compound 47 ligand–protein interaction diagram.

The MD simulation revealed that a stable ligand–protein complex was maintained throughout the complete simulation (Fig. 8). The lowest energy poses of compound 47 bind almost identically to a narrow channel formed by residues of K-RAS4B^allo^, SWI (Val29, Glu31, Tyr32, Asp33, Thr35, Ile36, Tyr40), and SWII (Gln61, Tyr64, Ser65, Ala66, Met67) as well as the residues Gln683, Pro684, Leu687, Met726, Lys728, and Trp729 of the SOS^Rem^ domain (Fig. 7c and d). The phthalic acid moiety is accommodated in a small lipophilic pocket, formed by RAS-residues of switch I, with the two methoxy groups located close to the hydroxy groups of the GTP ribose (Fig. 7d). The phthalic acid displaces two water molecules from the GTP·Mg^2+^ complex and is tightly anchored to its binding site by strong ionic interactions of Mg^2+^ with the negatively charged carboxylate anion and the *ortho*-ester carbonyl group (Fig. 7c). The octahedral coordination of the Mg^2+^ ion, as well as the interactions with the β- and γ-phosphate groups, are not affected by the binding of compound 47, in contrast to Ser17 and especially Thr35, which are displaced from their original positions by the aromatic ring. Further stabilization of the phthalic acid is achieved through water-mediated hydrogen bonds with Asp13, Asp30 and Glu31, as well as through a lipophilic interaction with Tyr32 (Fig. 8). The hydrocarbon scaffold of the oleanolic acid derivative 47 is additionally anchored by the 17-carboxy group, which forms strong water-mediated hydrogen bonds with the SOS^Rem^ residues Lys727, and especially Lys728, as well as a direct hydrogen bond with Met726 (Fig. 8). Apparently, the monophthalates of oleanolic acid and related pentacyclic triterpenes act as a molecular glue that stabilizes the K-RAS4B^allo^:SOS^Rem^ complex.

At first glance, the proposed mode of action appears counter-intuitive, as stabilization of a K-RAS4B^allo^:SOS^Rem^ complex should accelerate the rate of GDP exchange and thereby increase the level of active (GTP)RAS. However, the activation of SOS mediated by K-RAS4B^allo^ and the subsequent exchange of GDP/GTP in K-RAS4B^cat^ represents a complex feedback loop that depends on several conformational changes in all three proteins involved. The first step in GDP/GTP exchange is the insertion of the helical hairpin from helices αH and αI of SOS^Cdc25^, which leads to move-ments of the switch I and II regions that open the nucleotide binding site and facilitate the release of GDP.^58^

The nucleotide-free K-RAS4B:SOS complex is stabilized by the side chain of ^SOS^Leu938, which prevents Mg^2+^ binding, and ^SOS^Glu942, which overlaps with the binding site of the α-phosphate. It is also worth noting that during the entire MD simulation time of 500 ns, no significant conformational changes occurred within these two critical amino acids and all K-RAS4B^kat^ residues (Phe28, Val29, Asp30, Asn116, Lys117, Asp119, Ala146) that interact with guanosine and are thus important for GTP reentry.

**Fig. 9:**
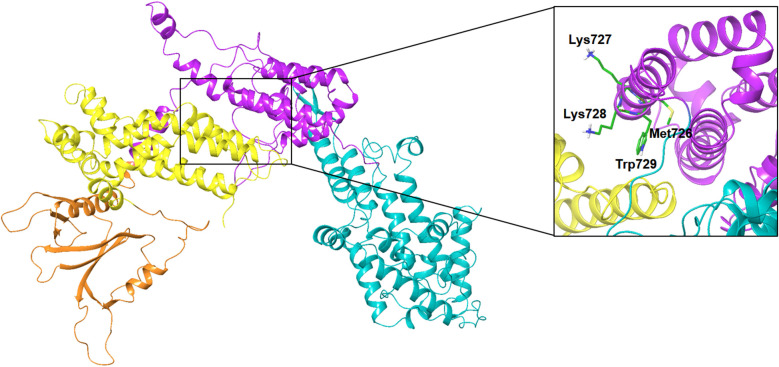
Autoinhibition of SOS-DH-PH. PDB code: 1XD4, green carbons: residues involved in compound 47 binding, PH domain: orange color, DH domain: yellow color, Rem domain: magenta color, CDC25 domain: cyan color, EPS8 linker: red color.

In contrast, the activation process of SOS upon binding of K-RAS4B^allo^ is less well understood. For H-RAS, it has been shown that the formation of the ternary complex (GppNHp)H-RAS^Y64A^(allo):SOS:H-RAS^wt^ induces a profound conformational change of the Rem domain relative to the CDC25 domain, which allosterically enhances the interactions between the switch I region of nucleotide-free K-RAS4B^cat^ and SOS, thereby promoting the rate of nucleotide exchange.^3^ Furthermore, it was shown that a SOS protein containing the Rem-domain preceding DH (Dbl homology) and PH (Pleckstrin homology) domains is autoinhibited by the DH domain, which packs against the face of the Rem domain (Fig. 9).^59,60^ The SOS^Rem^ residues 724–729, which are an essential part of the triterpene binding pocket, are completely occluded by the DH domain in the autoinhibited SOS state (Fig. 9). Our results suggest that oleanolic acid derivative 47 and related pentacyclic triterpenes act in a similar manner by blocking the same residues essential for the activation of SOS, thereby stabilizing an unproductive K-RAS4B^allo^:SOS complex or preventing the formation of highly active SOS states.^5^ The limited selectivity of several monophthalate-modified pentacyclic triterpenes can also be explained by the proposed mode of action, since oncogenic (GTP)K-RAS4B proteins activate SOS by binding to the allosteric site to a similar extent as the wild-type form.^61^

## Conclusion

Monophthalate derivatives of the natural products oleanolic acid, betulinic acid, and related pentacyclic triterpenes represent a new class of low micromolar GDP/GTP exchange inhibitors, which disrupt specifically downstream signalling *via* the RAS-PI3K-PDK1-AKT pathway in K-RAS4B mutant cells. What makes these compounds particularly interesting is their activity against the G12V mutant, which is the most difficult to target of all common K-RAS4B mutations. Our binding model implies that the monophthalate moiety forms an octahedral complex with Mg^2+^ in (GTP)K-RAS4B^allo^ by replacing two water molecules. The Mg^2+^ bound pentacyclic triterpenes then act as a molecular glue that stabilizes an unproductive K-RAS4B^allo^:SOS^Rem^ complex or prevents the formation of highly active SOS states.

Disrupting the K-RAS4B^allo^:SOS^Rem^ interaction appears an attractive new strategy for combating RAS-related cancers. However, the potential of this mode of action for the development of mutant-selective K-RAS4B therapeutics needs to be further investigated as a prerequisite for effective clinical translation.

## Experimental section

### Computational methods

#### *In silico* docking

All calculations were done with the Schrödinger molecular modeling suite (Maestro version 11.5). For *in silico* dockings all ligands were prepared with the LigPrep tool. Protonation states and tautomers were calculated for pH ± 2 using Epik. The raw PDB files of protein crystal structures were optimized for *in silico* dockings with the protein preparation wizard. Missing amino acid side chains and loops were added with the software tool Prime. Protonation and tautomerization states were optimized with Epik for pH ± 1. After removal of water molecules beyond 5 Å distance from heavy atoms, an energy minimization of the protein to RMSD ± 0.3 Å with OPLS2005 was performed. For *in silico* dockings Glide, a component of the Schrödinger molecular modeling package (2018), was used with standard settings in XP (extraprecision) mode.

#### MM-GBSA calculations

MM-GBSA calculations were performed with Prime, a component of the Schrödinger molecular modeling package (version 2018). In a first step, an ensemble of energetically favoured conformers of the ligand was generated by a mixed torsional/low mode sampling with the macromodel conformational search function (CSearch, standard parameters, max. number of steps: 100). The conformational search provided between 60–90 different conformers, which were subsequently preoriented in the binding-pocket by superposition. Conditions for all MM-GBSA calculations were: solvation model, VSGB; force field, OPLS2005; protein flexibility: 0 Å.

#### Molecular dynamics simulation

The molecular dynamics (MD) simulation of compound 47 was performed with Desmond, a component of the Schrödinger molecular modeling package. Conditions were: box shape, orthorhombic; solvent model, SPC; solvent, 0.15 M NaCl solution; simulation time, 500 ns; recording interval, 250 ps; temp, 300 K; pressure, 1.01325 bar.

### Biological testing

#### Nucleotide exchange assay

Inactive, GDP-bound K-RAS is incubated with SOS1 and GTP. K-RAS is transferred to its active GTP-bound state which leads to a release of GDP. The K-RAS bound GTP is hydrolysed to GDP even in the absence of the corresponding GAP protein. In the GDP GloBioluminescent GDP detection assay for glycosyltransferases (Promega, Madison, WI, USA) used, GDP is converted to ATP which can be quantified using a luciferase/luciferin reaction. The resulting luminescence signal is then measured with a suitable microplate reader. This assay was developed for K-RAS wild type protein as well as for the G12D and G12V mutant.

For each sample, 5 μL K-RAS working solution in assay buffer (50 mM HEPES pH 7.5, 4 mM MgCl_2_, 2 mM EGTA, 0.01% Brij35, 1 mM TCEP) were transferred into a suitable assay plate (*e.g.* Greiner #784075). The test compound was added with an echoacoustic dispenser (Beckman Coulter, Brea, CA, USA) in a concentration range from 3000 μM to 3 μM (8-point dilution). After addition of the test compound 5 μL of SOS1-GTP mix in assay buffer were added. The reaction mixture was incubated overnight at room temperature followed by the addition of 10 μL GPD detection reagent. After a second incubation period of 1 h at room temperature the luminescence signal was measured with an Envision spectrophotometer (PerkinElmer, Waltham, MA, USA). IC_50_ values were determined from the sigmoidal dose response curves with the software Quattro Workflow (Quattro GmbH, Munich, Germany).

To filter out compounds which interfere with the nucleotide exchange assay independent from K-RAS and SOS1, a control assay was developed. For this, GDP was titrated and detected with the GDP Glo bioluminescent GDP detection assay for glycosyltransferases (Promega, Madison, WI, USA) to generate a luminescence signal comparable to the positive control of the nucleotide exchange assay. Compounds were checked for assay interference by performing dose response curves in the same concentration range as for the nucleotide exchange assay. For each sample, 10 μL GDP (125 nM final assay concentration) in assay buffer (50 mM HEPES pH 7.5, 4 mM MgCl_2_, 2 mM EGTA, 0.01% Brij35, 1 mM TCEP) was transferred into a suitable assay plate (*e.g.* Greiner #784075). The test compound was added with an Echo acoustic dispenser (Beckman Coulter, Brea, CA, USA) in a concentration range from 3000 μM to 3 μM (8-point dilution). The reaction mixture was incubated over night at room temperature followed by the addition of 10 μL GPD detection reagent. After a second incubation period of 1 h at room temperature the luminescence signal was measured with an Envision spectrophotometer (PerkinElmer, Waltham, MA, USA). IC_50_ values were determined from the sigmoidal dose response curves with the software Quattro Work-flow (Quattro GmbH, Munich, Germany).

#### HTRF Akt/Erk phosphorylation assay

SNU-1 cells harbouring the K-RASG12D mutation (primary carcinoma of the stomach), were grown in RPMI medium with 10% FBS (fetal bovine serum) and 1% l-Gln. Cells were plated in white small volume cell culture 384 well microplates (Greiner BioOne GmbH) at a density of 50.000 cells per well/6 μL in RPMI medium with 1% l-glut (serum-free medium). The plated cells were placed in a high humidity incubator (37 °C, 5% CO_2_ and 90% humidity) to avoid evaporation of the small amount of medium for 3 hours before the compounds were added. With the Echo520 liquid handler the cells were treated with a 3-fold 8-point serial dilution of the compounds, with a top final concentration of 330 μM and a final DMSO concentration of 1%. After compound transfer, cells were incubated for 1 hour in the high humidity incubator before the cells were treated with 3 μL of 9 nM EGF diluted in serum-free medium (final EGF concentration in the well 3 nM) for 15 minutes. The lysis buffer from the Phospho-ERK 1/2 (Thr202/Tyr204)- and the Total ERK1/2 LANCE Ultra TR-FRET Cellular Detection Kit (PerkinElmer Inc.) was prepared to a 4-fold concentration in water. Then 3 μL of the lysis buffer were added to each well and the plates were shaken at 300 rpm for 30 minutes at room temperature. During this incubation the antibody detection mix was prepared according to the manufacturer's protocol. In the final step 3 μL of antibody detection mix were added to each well. The plates were sealed with an aluminium sticky foil and incubated over night at room temperature for 20 hours. The detection was performed with the EnVision2104 Multilabel Reader.

#### NanoBRET target engagement assay

The NanoBRET® TE Intracellular RAS Assay uses NanoBiT® Technology, a structural complementation system comprised of a large luciferase subunit (LgBiT) and a small complementary peptide (SmBiT(q)). Specifically, a fusion protein of LgBiT and full-length RAS is co-expressed with a fusion protein of SmBiT(q) and full-length RAS in cells. When the RAS proteins interact and form multimers, the LgBiT and SmBiT(q) subunits come together to form an active luciferase enzyme that serves as the BRET donor. The energy acceptor is a cell-permeable fluorescent NanoBRET® tracer that binds specifically to RAS. Addition of compounds that bind to RAS displaces the tracer and results in a dose-dependent loss of BRET signal. HEK239T cells were transfected with LgBiT-KRAS2B (G12D) and SmBiT-KRAS2B (G12D) Fusion Vectors using a standard Mirus LT1 TransIT Transfection Reagent protocol. 9 μL cells (2160 cells per well) were transferred to a suitable assay plate (*e.g.* Greiner #784080) and incubated for 24 h at 37 °C and 5% CO_2_. Compounds were added in a suitable concentration range *e.g.* from 100 μM to 0.025 μM (8-point dilution) *via* an acoustic dispenser (BeckmanCoulter, Brea, CA, USA) equipped with Echo Dose Response software. After mixing the plate for 1 min and incubation at room temperature for 15 min 1 μL of NanoBRET TE RAS Tracer 01 (final concentration 1 μM) was added followed by another mixing step. After 2 h incubation (37 °C, 5% CO_2_) and 15 min at room temperature 5 μL of NanoBRET™ Nano-Glo® substrate was added followed by a mixing step. After incubation for 10–30 min at room temperature in the dark the BRET signal was measured at 460 nm and 645 nm emission with an Envision microplate reader (Revvity, Waltham, MA, USA). IC_50_ values were determined from the sigmoidal dose response curves with Scigilian Analyze software (Scigilian, Montreal, Canada).

#### HTRF K-RAS^wt^:SOS protein–protein interaction assay

To test the compounds effect on the interaction of K-RAS and SOS1 proteins a commercial assay from Revvity (product code 64KRASWTPEG) was used. The K-RAS WT/SOS1 PPI assay includes tagged human recombinant partners (K-RAS WT and SOS1) and labelled anti-tag reagents for HTRF detection. Without an inhibitor, K-RAS WT loaded with GTP binds to SOS1, and the binding of each detection reagent to its tagged target generates an HTRF signal. In the presence of K-RAS WT/SOS1 inhibitors or GTP competitors, the HTRF signal decreases.

For every sample, 1.5 μL assay buffer and 3 μL of Tag2-SOS1 protein was transferred into a suitable assay plate (*e.g.* Corning #4513). Compound was added *via* Echo acoustic dispenser (BeckmanCoulter, Brea, CA, USA) in a suitable concentration range *e.g.* from 50 μM to 0.115 μM (8-point dilution). After an incubation step for 10 min at room temperature 3 μL Tag1-KRAS protein was added followed by 7.5 μL detection mix containing anti-Tag2 antibody labeled with Terbium cryptate and anti-Tag1 antibody labeled with XL665. After an incubation for 2 h at room temperature the HTRF signal was measured at 340 nm excitation, 665 nm and 615 nm emission with an Envision microplate reader (Revvity, Waltham, MA, USA). IC_50_ values were determined from the sigmoidal dose response curves with the Scigilian Analyze software (Scigilian, Monteral, Canada).

## Data availability

The data supporting the results of this study are provided in the article or have been included as part of the ESI† (experimental procedures and characterization of all compounds).

## Author contributions

J. S.: experimental design; G. E. B., F. K., S. K.: experimental work; J. S., G. E. B.: molecular modeling; J. S.; G. E. B.: manuscript writing and revision.

## Conflicts of interest

There are no conflicts of interest to declare.

## Supplementary Material

RA-015-D4RA08503E-s001
