# Supplementary material for: Monophthalates of betulinic acid and related pentacyclic triterpenes inhibit efficiently the SOS-mediated nucleotide exchange and impact PI3K/AKT signaling in oncogenic K-RAS4B proteins
Source: RSC Adv. 2025 Jan 10;15(2):883–95. doi: 10.1039/d4ra08503e (PMC11719397; doi:10.1039/d4ra08503e)
Supplement: RA-015-D4RA08503E-s001 [file RA-015-D4RA08503E-s001.pdf]

Supplementary Information (SI) for RSC Advances.
This journal is © The Royal Society of Chemistry 2025

Supporting Information

Monophthalates of betulinic acid and related pentacyclic triterpenes inhibit efficiently the SOS-mediated nucleotide exchange and impact PI3K/AKT signaling in oncogenic K-RAS4B proteins

Gerrit E. Benary[a], Frank Kilgenstein[a], Sascha Koller[a], Jürgen Scherkenbeck[a]*

Chemical Syntheses

Abbreviations: AcOH, acetic acid; Cyh, cyclohexane; DCM, dichloromethane; DCVC, dry column vacuum chromatography; DIPEA, N,N-diisopropylethylamine; DMAP, 4-dimethylaminopyridine; DMP, Dess-Martin periodinane; DMF, N,N dimethylformamide; DMSO, dimethyl sulfoxide; EDCI, 1-ethyl-3-(3-dimethyl-aminopropyl)carbodiimide; EtOAc, ethylacetate; MeCN, acetonitrile; MEMCl, 2-methoxyethoxymethyl chloride; TFA, trifluoroacetic acid; THF, tetrahydrofuran.

General: Reagents and solvents were purchased from commercial sources and used without further purification, unless otherwise stated. CH_2Cl_2 was dried with a MB-SPS-800 solvent purification system. MeOH was redistilled from magnesium turnings. DMF was degassed with helium before use to remove traces of dimethylamine. Reactions were stirred magnetically under an argon atmosphere unless otherwise stated. A freeze dryer, Zirbus GOT2000, was used for lyophilization of aqueous solutions. Microwave reactions were performed in a Cem Discover System microwave. Reactions under inert gas were performed according to standard Schlenk procedures. Reactions were monitored by TLC carried out on Macherey Nagel silica gel plates (60F-254) or Merk silica gel 60 RP-18 F254 plates using UV light. TLC plates were stained either with an aqueous solution of KMnO4, K2CO3, NaOH or an aqueous solution of $Ce(SO_4)_2$, $H_3PMo_{12}O_{40}$, H_2SO_4. Crude products were purified by column chromatography with pre-packed silica cartridges in different sizes (60 M Macherey-Nagel, particle size 40-64 µm, pore size 60Å or Interchim, PF-15SIHP or PF-30SIHP). The cartridges were used with a puriFlash®XS 420 system. Pre-packed cartridges (Interchim, PF-15C18AQ) in different sizes were used for purification by preparative reverse phase column chromatography. For preparative HPLC purification a Nucleodur (SP250/21, 100-5 C18 ec, Macherey-Nagel) column was used. Reverse phase column chromatography and preparative HPLC purification were performed with a puriflash® 4250 system (Interchim). ^{1}H and ^{13}C NMR spectra were recorded on Bruker Avance III 600 and Bruker Avance 400 spectrometers operating at 600 and 400 MHz (^{1}H) respectively and 151 and 101 MHz (^{13}C), respectively. IR spectra were recorded on a Bruker ALPHA FTIR spectrometer. The absorption bands are given in wave number (cm^{-1}). The intensities of the bands are categorized as strong (s), medium (m) or weak (w). HPLC measurements were performed with a Shimadzu Prominence-i LC-2030C 3D Plus coupled with a Shimadzu LCMS-2020. The following methods were used and are given for every Substance. **Method A:** HPLC-Column: Perfect Sil Target ODS-3 HD 5 µm 100x4.6 mm, 90% H_2O (5 mM NH_4OAc) to 90% MeCN, 24 min, 1.5 mL/min, 200-800 nm; ESI: positive and/or negative. **Method B:** HPLC-Column: Shim-packGISS C18 1.9 µm 50x2.1 mm or Raptor ARC-18 1.9 µm 50x2.1 mm; 95% H_2O (0.1% HCOOH) to 95% MeCN (0.1% HCOOH); 16 min, 400 µL/min; 200-800 nm; ESI: positive and/or negative. **Method C:** HPLC-Column: Shim-packGISS C18 1.9 µm 50x2.1 mm or Raptor ARC-18 1.9 µm 50x2.1 mm; 50% H_2O (0.1% HCOOH) to 95% MeCN (0.1% HCOOH); 16 min, 400 µL/min; 200-800 nm; ESI: positive and/or negative. **Method D:** HPLC-Column: Knauer Eurospher II C18 1.8 µm 100x2mm; 50% H_2O (0.1% HCOOH) to 95% MeCN (0.1% HCOOH); 16 min, 300 µL/min; 200-800 nm; ESI: positive and/or negative. Accurate mass determinations were measured with a Bruker micrOTOF (ESI-MS)$^+$ Agilent 1100 Series mass spectrometer.

General procedures for the preparation of the phthalate derivatives of pentacyclic triterpenes

The phthalate-derivatives were synthesized according to the procedure developed by Kvasnica et al.[1]

Procedure A: The pentacyclic triterpene, substituted phthalic anhydride and DMAP were dissolved in pyridine and stirred in a round-bottom flask. After completion of the reaction, the solution was diluted with EtOAc and washed twice with a 1M aqueous solution of HCl and twice with sat. NaCl-solution. The organic layer was dried over Na_2SO_4 and concentrated under reduced pressure. The resulting solid was purified via column chromatography.

Procedure B: The pentacyclic triterpene was solved in pyridine and placed in a microwave reaction vial. The substituted phthalic anhydride was added and the reaction was heated up via microwave irradiation to 100 °C. After finishing the reaction, the solution was diluted with EtOAc and washed twice with a 1M aqueous solution of HCl. The organic layer was concentrated under reduced pressure and the resulting solid purified via column chromatography.

*(1R,3aS,5aR,5bR,7aR,9S,11aR,11bR,13aR,13bR)-9-Acetoxy-5a,5b,8,8,11a-pentamethyl-1-(prop-1-en-2-yl)-icosahydro-3aH-cyclopenta[a]chrysene-3a-carboxylic acid (**8**).*[2] A mixture of betulinic acid (100.0 mg, 0.22 mmol, 1.00 eq), acetic anhydride (34.7 µl, 0.32 mmol, 1.50 eq), DMAP (2.7 mg, 0.02 mmol, 0.10 eq), and DIPEA (0.261 ml, 1.53 mmol, 7.00 eq) in THF (5.0 ml) was refluxed for five hours. Water (2.0 ml) was added and the solution was stirred for an additional hour. The solution was extracted three times with $CHCl_3$ (10.0 ml) and the organic layer was dried over Na_2SO_4. After solvent evaporation the crude product was purified by silica gel chromatography (SiO_2, Cyh:EtOAc 1:1, silica gel: 23 g) to yield product **8** (60.0 mg, 55%). HPLC (method B): t_R = 13.2 min, purity 95%. ^{1}H-NMR ($CDCl_3$, 600 MHz) δ: 4.69 (s, 1H), 4.56 (s, 1H), 4.36 (dd, 1H, J = 11.7, 4.6 Hz), 2.96 (td, 1H, J = 10.8, 5.1 Hz), 2.26-2.19 (m, 1H), 2.14-2.08 (m, 1H), 1.99 (s, 3H), 1.83-1.76 (m, 2H), 1.65 (s, 1H), 1.64-1.57 (m, 3H), 1.56-1.45 (m, 3H), 1.46-1.38 (m, 3H), 1.38-1.30 (m, 6H), 1.21-1.07 (m, 2H), 1.03-0.96 (m, 1H), 0.94 (s, 3H), 0.93-0.88 (m, 1H), 0.87 (s, 3H), 0.90 (s, 3H), 0.79 (s, 6H), 0.78-0.69 (m, 1H). ^{13}C-NMR ($CDCl_3$, 150 MHz) δ: 177.2, 170.0, 150.3, 109.5, 79.8, 54.6, 49.6, 48.5, 46.6, 42.0, 40.2, 37.8, 37.5, 37.3, 36.6, 36.3, 33.7, 31.6, 30.0, 29.1, 27.7, 25.0, 23.3, 21.0, 20.4, 19.0, 17.7, 16.4, 15.9, 15.6, 14.4. IR (neat) v_{max}/cm^{-1} 2940 (s), 2864 (w), 1734 (s), 1690 (s), 1463 (w), 1365 (m), 1245 (s), 1012 (w), 979 (m), 924 (w), 883 (m), 797 (w). HRMS-ESI m/z calcd for $C_{32}H_{50}O_4$ $(M+Na)^+$: 521.3601, found 521.3600.

*(1R,3aS,5aR,5bR,7aR,9S,11aR,11bR,13aR,13bR)-9-(Benzoyloxy)-5a,5b,8,8,11a-pentamethyl-1-(prop-1-en-2-yl)icosahydro-3aH-cyclopenta[a]chrysene-3a-carboxylic acid (**9**).*[3] Betulinic acid (200.0 mg, 0.44 mmol, 1.00 eq) and DMAP (5.35 mg, 0.04 mmol, 0.10 eq) were dissolved in pyridine (5.0 ml). Benzoyl chloride (60.55 µl, 0.53 mmol, 1.2 eq) was added and the mixture was stirred for 5 days at 100 °C. The solution was diluted with DCM (20.0 ml) and washed with saturated NaCl solution (2x 20.0 ml), saturated $NaHCO_3$ solution (2x 20.0 ml), and saturated NaCl solution (2x 20.0 ml). The organic layer was dried over Na_2SO_4 and the solvent was removed under reduced pressure. The pure compound **9** (102.8 mg, 42%) was obtained after recrystallization from $MeCN/H_2O$ (9:1). HPLC (method B): t_R = 14.5 min, purity >95%. ^{1}H-NMR ($CDCl_3$, 600 MHz) δ: 7.97-7.94 (m, 2H), 7.66-7.63 (m, 1H), 7.53 (dd, 2H, J = 7.80, 7.80 Hz), 4.71-4.68 (s, 1H), 4.65-4.59 (m, 1H), 4.58 (s, 1H),), 2.95 (td, 1H, J = 10.7, 5.0 Hz), 2.27-2.20 (m, 1H), 2.14-2.09 (m, 1H), 1.86-1.77 (m, 2H), 1.75-1.66 (m, 2H) 1.65 (s, 1H), 1.64-1.61 (m, 1H), 1.55-1.50 (m, 1H), 1.50-1.36 (m, 8H), 1.35-1.28 (m, 3H), 1.22-1.14 (m, 1H), 1.14-1.07 (m, 1H), 1.07-0.98 (m, 2H), 0.96 (s, 3H), 0.95 (s, 3H), 0.89 (s, 3H), 0.85 (s, 6H), 0.81-0.78 (m, 1H). ^{13}C-NMR ($CDCl_3$, 151 MHz) δ: 177.1, 165.2, 150.2, 138.1, 130.1, 128.9, 128.6, 109.5, 80.8, 55.3, 54.5, 49.5, 48.4, 46.4, 41.9, 40.1, 37.6, 37.4, 36.5, 36.2, 33.6, 31.6, 30.0, 29.1, 27.6, 24.9, 23.2, 20.3, 18.8, 17.6, 16.4, 15.7, 15.6, 14.2. IR (neat) v_{max}/cm^{-1} 2939 (m), 2869 (w), 1717 (m), 1694 (m), 1451 (w), 1270 (s), 1113 (m), 1026 (w), 970 (w), 888 (w), 710 (s). HRMS-ESI m/z calcd for $C_{37}H_{52}O_4$ $(M+Na)^+$: 583.3758, found 583.2757.

*Methyl (1R,3aS,5aR,5bR,7aR,9S,11aR,11bR,13aR,13-bR)-9-hydroxy-5a,5b,8,8,11a-pentamethyl-1-(prop-1-en-2-yl)icosa-hydro-3aH-cyclopenta[a]chrysene-3a-carboxylate (**10**).*[4] A solution of betulinic acid (1.0 g, 2.19 mmol, 1.00 eq) in THF (8.0 ml) was added to a suspension of NaH (60%, 157.7 mg, 6.57 mmol, 3.00 eq) in THF (4.5 ml) and stirred for 30 minutes at room temperature under an argon atmosphere. Then, MeI (0.818 ml, 13.14 mmol, 6.00 eq) was added and stirring continued for 17 h at 65 °C. The solvent was evaporated under reduced pressure and the remaining residue was dissolved in EtOAc (50 ml). The organic layer was washed three times with saturated NH_4Cl solution (20.0 ml) and H_2O (20.0 ml) and then dried over Na_2SO_4. After evaporation of the solvent *in vacuo*, the crude product was purified by silica gel chromatography (Cyh:EtOAc 19:1, silica gel: 23 g) to give compound **10** (818.2 mg, 79%). HPLC (method C): t_R = 10.5 min, purity >95%. ^{1}H-NMR ($CDCl_3$, 400 MHz) δ: 4.74-4.72 (m, 1H), 4.61-4.59 (m, 1H), 3.66 (s, 3H), 3.18 (dd, 1H, J = 11.2, 5.1 Hz), 2.99 (td, 1H, J = 11.0, 4.6 Hz), 2.25-2.15 (m, 2H),), 1.93-1.84 (m, 2H), 1.73-1.69 (m, 1H), 1.68 (s, 1H), 1.67-1.64 (m, 1H), 1.63-1.54 (m, 3H), 1.51-1.48 (m, 2H), 1.40-1.35 (m, 6H), 1.30-1.23 (m, 2H), 1.16-1.08 (m, 1H), 1.05-0.99 (m, 1H), 0.96 (s, 6H), 0.91 (s, 3H), 0.90-0.84 (m, 1H), 0.82 (s, 3H), 0.75 (s, 3H), 0.70-0.65 (m, 1H). ^{13}C-NMR ($CDCl_3$, 101 MHz) δ: 176.8, 150.5, 109.7, 79.2, 56.7, 55.5, 51.4, 50.7, 49.7, 47.1, 42.6, 40.9, 39.0, 38.9, 38.5, 37.4, 37.1, 34.5, 32.4, 30.8, 29.9, 28.1, 27.6, 25.7, 21.1, 19.5, 18.5, 16.3, 16.1, 15.5, 14.9. IR (neat) v_{max}/cm^{-1} 3538 (w), 3455 (w), 2939 (m), 2869 (w), 1718 (s), 1699 (s), 1640 (w), 1443 (m), 1192 (w), 1083 (w), 886 (s), 782 (w). HRMS-ESI m/z calcd for $C_{31}H_{50}O_3$ $(M+Na)^+$: 493.3652, found 493.3648.

*Methyl (1R,3aS,5aR,5bR,7aR,9S,11aR,11bR,13aR,13-bR)-9-methoxy-5a,5b,8,8,11a-pentamethyl-1-(prop-1-en-2-yl)-icosahydro-3aH-cyclopenta[a]chrysene-3a-carboxylate (**11**).* Compound **11** (155.0 mg, 15%) was obtained as a byproduct in the synthesis of compound **10** and was used without further characterization for the synthesis of compound **15**. HPLC (method C): t_R = 14.4 min, purity >95%.

*((1R,3aS,5aR,5bR,7aR,9S,11aR,11bR,13aR,13bR)-9-Hydroxy-5a,5b,8,8,11a-pentamethyl-1-(prop-1-en-2-yl)icosahydro-1H-cyclopenta[a]chrysene-3a-carbo-nyl)glycine (**12**).*[5] At 0 °C a 0.25 M aqueous NaOH solution (30.0 ml) was added to a solution of starting material **21** (400.0 mg, 0.74 mmol, 1.00 eq) in THF (10.0 ml) and H_2O (15.0 ml). The reaction was stirred for four days at room temperature. Then, the solution was concentrated under reduced pressure and diluted with EtOAc (20.0 ml). The layers were separated and the organic layer was washed twice with H_2O (10.0 ml), sat. NaCl solution (10.0 ml), sat. $NaHCO_3$ solution (10.0 ml), sat. NaCl solution (10.0 ml), and H_2O (10.0 ml). The organic layer was dried over Na_2SO_4. After filtration and removal of the solvent, the crude product was purified by silica gel chromatography (Cyh:EtOAc 1:2, silica gel: 32 g) to yield pure compound **12** (293.3 mg, 77%). HPLC (method B): t_R = 10.5 min, purity >95%. ^{1}H-NMR ($CDCl_3$, 600 MHz) δ: 7.86 (s, 1H), 4.64 (s, 1H), 4.24 (s, 1H), 3.70-3.58 (m, 2H), 3.01-2.93 (m, 2H), 2.53-2.51 (m, 1H), 2.18-2.11 (m, 1H), 1.87-1.80 (m, 1H), 1.79-1.71 (m, 1H, 1.62 (s, 1H), 1.60-1.48 (m, 3H), 1.47-1.39 (m, 5H), 1.37-1.26 (m, 5H), 1.25-1.20 (m, 2H), 1.17-1.09 (m, 1H), 1.07-0.95 (m, 1H), 0.91 (s, 3H), 0.90-0.88 (m, 1H), 0.86 (s, 3H), 0.84 (s, 3H), 0.82-0.78 (m, 1H), 0.75 (s, 3H), 0.65 (s, 3H), 0.63-0.58 (m, 1H). ^{13}C-NMR ($CDCl_3$, 151 MHz) δ: 176.1, 171.6, 150.9, 109.1, 76.8, 54.9, 54.8, 46.1, 41.9, 40.9, 38.5, 38.3, 37.5, 36.7, 36.6, 34.9, 32.2, 30.2, 28.8, 28.1, 27.2, 25.2, 20.5, 19.0, 17.9, 15.9, 15.8, 15.8, 14.3. IR (neat) v_{max}/cm^{-1} 3594 (w), 2933 (s), 2867 (m), 1737 (m), 1638 (m), 1529 (w), 1447 (w), 1376 (w), 1194 (s), 1029 (m), 1007 (w), 983 (w), 880 (s),727 (w). HRMS-ESI m/z calcd for $C_{32}H_{50}NO_4$ $(M-H)^-$: 512.3745, found 512.3740.

*3H-[1,2,3]Triazolo[4,5-b]pyridin-3-yl (1R,3aS,5aR,5-bR,7a-R,9S,11aR,11bR,13aR,13bR)-9-hydroxy-5a,5b,-8,8,11a-penta-methyl-1-(prop-1-en-2-yl)icosahydro-3aH-cyclopenta[a]-chrysene-3a-carboxylate (**13**).* The active ester **13** (8.7 mg, 7%) was isolated as a byproduct in the synthesis of compound **21**. HPLC (method B): t_R = 13.4 min, purity >99%. ^{1}H-NMR ($CDCl_3$, 400 MHz) δ: 8.83 (d, 1H, J = 4.5 Hz), 8.71 (d, 1H, J = 8.5 Hz), 4.71 (s, 1H), 4.60 (s, 1H), 4.24 (d, 1H, J = 5.1 Hz), 3.03-2.99 (m, 1H), 2.87-2.78 (m, 1H), 2.55-2.51 (m, 1H), 2.36-2.22 (m, 1H) 2.14-2.02 (m, 1H), 2.03-1.88 (m, 2H), 1.88-1.74 (m, 3H), 1.69 (s, 1H), 1.65-1.58 (m, 1H), 1.57-1.50 (m, 2H), 1.49-1.41 (m, 3H), 1.41-1.32 (m, 5H), 1.17-1.06 (m, 1H), 1.02 (s, 3H), 1.01-0.94 (m, 1H), 0.92 (s, 3H), 0.89 (s, 3H), 0.87-0.82 (m, 1H), 0.75 (s, 3H), 0.66 (s, 6H). ^{13}C-NMR ($CDCl_3$, 151 MHz) δ: 171.6, 152.6, 149.1, 140.1, 134.5, 129.8, 121.7, 110.3, 76.7, 56.5, 54.8, 49.8, 49.0, 46.4, 42.1, 40.3, 38.5, 38.2, 38.1, 36.7, 35.7, 33.8, 30.5, 29.7, 29.4, 28.0, 27.1, 24.9, 20.3, 18.9, 17.9, 15.9, 15.7, 15.5, 14.4. IR (neat) v_{max}/cm^{-1} 2936 (s), 2869 (w), 1749 (w), 1437 (m), 1376 (m), 1362 (m), 1243 (m), 1105 (m), 1062 (m), 1008 (s), 993 (s), 935 (s), 801 (m), 769 (s), 719 (s). HRMS-ESI m/z calcd for $C_{35}H_{50}N_4O_3$ $(M+Na)^+$: 597.3775, found 597.3776.

*(1R,3aS,5aR,5bR,7aR,9S,11aR,11bR,13aR,13bR)-9-((2-Carboxybenzoyl)oxy)-5a,5b,8,8,11a-pentamethyl-1-(1-oxopropan-2-yl)icosahydro-3aH-cyclopenta[a]-chrysene-3a-carboxylic acid (**14**).* Compound **19** (60.0 mg, 0.10 mmol, 1.00 eq) was dissolved in DCM (1.0 ml). A saturated $NaHCO_3$ solution (0.01 ml) and *m*CPBA (34.2 mg, 0.20 mmol, 2.00 eq) were added at 0 °C. The mixture was stirred for 3 hours at 0 °C, followed by solvent evaporation under reduced pressure. The crude product was purified by column chromatography to obtain product **14** (12.3 mg, 20%) as a mixture of diastereomers. HPLC (method B): t_R = 11.3 min, purity >99% (d.r.: 2:1). ^{1}H-NMR ($CDCl_3$, 600 MHz) δ: 9.87 (d, 1H, J = 1.7 Hz), 9.68 (s, 1H), 7.90 (m, 1H), 7.90 (dd, 1H, J =7.6, 1.6 Hz), 7.63-7.57 (m, 2H), 4.76 (dd, 1H, J = 11.8, 4.3 Hz), 3.02-2.98 (m, 1H), 2.68-2.67 (m, 1H), 2.62-2.59 (m, 1H), 2.48-2.45 (m, 1H), 2.35-2.24 (m, 2H), 0.89-1.99 (m, 41H). ^{13}C-NMR ($CDCl_3$, 151 MHz) δ: 207.0, 182.8, 172.6, 167.9, 133.7, 132.1, 130.9, 130.5, 129.9, 129.2, 83.3, 56.9, 56.6, 55.6, 50.1, 49.9, 49.3, 48.3, 42.7, 40.9, 38.5, 38.2, 37.3, 37.1, 37.0, 34.4, 31.9, 29.8, 28.2, 27.5, 26.7, 25.8, 24.5, 23.1, 21.1, 18.3, 16.8, 16.8, 16.4, 14.6, 7.0. IR (neat) v_{max}/cm^{-1} 2945 (s), 2873 (w), 1698 (s), 1454 (w), 1392 (w), 1378 (w), 1288 (s), 1194 (w), 1129 (w), 1075 (w), 966 (w), 911 (w), 734 (m). HRMS-ESI m/z calcd for $C_{38}H_{51}O_7$ $(M-H)^-$: 619.3640 found 619.3642.

*Methyl (1R,3aS,5aR,5bR,7aR,9S,11aR,11bR,13aR,13-bS)-1-acetyl-9-methoxy-5a,5b,8,8,11a-pentamethyl-icosahydro-3aH-cyclopenta[a]chrysene-3a-carboxylate (**15**).* Ozone gas was passed through a solution of starting material **11** (100.0 mg, 0.21 mmol, 1.00 eq) in a 4:1 mixture of MeOH/DCM (10.0 ml) for 2 h at -78 °C. Excess of ozone was removed by passing N_2 gas through the reaction vessel until the blue colour had disappeared. The solution was quenched with dimethyl sulfide

(17 µl, 0.23 mmol, 1.10 eq) followed by removal of the solvent under reduced pressure. The crude product was purified by silica gel column chromatography (Cyh:EtOAc 19:1, silica gel: 23 g) to afford the product **15** (21.9 mg, 22%). HPLC (method C): t_R = 12.9 min, purity >95%. ^{1}H-NMR ($CDCl_3$, 400 MHz) δ: 3.66 (s, 3H), 3.34 (s, 3H), 3.25 (dd, 1H, J = 11.3, 4.6 Hz), 2.28-2.21), 2.17 (s, 3H), 2.10 (dd, 1H, J = 11.3 Hz), 2.05-1.93 (m, 2H), 1.91-1.82 (m, 1H), 1.79-1.66 (m, 3H), 1.43-1.19 (m, 10H), 1.19-1.15 (m, 1H), 1.09-1.02 (m, 2H), 0.98 (s, 3H), 0.93 (s, 3H), 0.88 (s, 3H), 0.86-0.82 (m, 1H), 0.80 (s, 3H), 0.72 (s, 3H), 0.70-0.63 (m, 1H). ^{13}C-NMR ($CDCl_3$, 101 MHz) δ: 212.3, 176.3, 80.4, 57.3, 56.2, 55.7, 51.2, 51.0, 50.2, 49.3, 42.0, 40.4, 38.6, 38.4, 37.2, 37.0, 36.4, 34.0, 31.3, 30.0, 29.5, 28.1, 27.8, 27.1, 22.0, 20.7, 18.0, 15.9, 15.9, 15.7, 14.5. IR (neat) v_{max}/cm^{-1} 2947 (s), 2867 (w), 2847 (w), 1724 (s), 1708 (s), 1449 (m), 1353 (m), 1135 (s), 1098 (s), 974 (m), 816 (w). HRMS-ESI m/z calcd for $C_{31}H_{50}O_4$ $(M+Na)^+$: 509.3601, found 509.3598.

Methyl (1R,3aS,5aR,5bR,7aR,9S,11aR,11bR,13aR,13-bS)-9-acetoxy-1-acetyl-5a,5b,8,8,11a-pentamethylico-sahydro-3aH-cyclopenta[a]chrysene-3a-carboxylate (16).[6] **Step1:** To a solution of the starting material **10** (70.8 mg, 0.15 mmol, 1.00 eq) and DMAP (1.8 mg, 0.02 mmol, 0.10 eq) in dry THF (3.0 ml) acetic anhydride (28.0 µl, 0.30 mmol, 2.00 eq) and DIPEA (0.18 ml, 1.05 mmol, 7.00 eq) were added. After refluxing for 5 h, the solvent was removed under reduced pressure. The residue was dissolved in $CHCl_3$ (10.0 ml) and washed three times with H_2O (5.0 ml). The organic layer was dried over Na_2SO_4 followed by solvent evaporation *in vacuo* to obtain the pure intermediate (70.0 mg, 91%). HPLC (method C): t_R = 14.0 min, purity >99%. ^{1}H-NMR (acetone-d_6, 600 MHz) δ: 4.76 (d, 1H, J = 2.4 Hz), 4.63 (t, 1H, J = 2.0 Hz), 4.47 (dd, 1H, J = 11.3, 5.2 Hz), 3.69 (s, 3H), 3.05 (td, 1H, J = 10.8, 5.1 Hz), 2.36-2.27 (m, 1H), 2.28-2.21 (m, 1H), 2.08-2.02 (m, 6H), 1.96-1.84 (m, 2H), 1.80-1.68 (m, 5H), 1.70-1.59 (m, 2H), 1.59-1.36 (m, 8H), 1.34-1.26 (m, 1H), 1.22-1.16 (m, 1H), 1.13 (td, 1H, J = 13.0, 4.6 Hz), 1.05 (s, 4H), 0.97 (s, 3H), 0.92 (s, 3H), 0.90 (d, 1H, J = 2.0 Hz), 0.88 (s, 3H), 0.88 (s, 4H). ^{13}C-NMR (acetone-d_6, 151 MHz) δ: 174.6, 169.2, 149.0, 107.7, 79.0, 54.8, 53.7, 49.2, 48.8, 47.7, 45.5, 40.7, 39.0, 36.6, 36.6, 36.0, 35.4, 34.9, 32.5, 30.2, 28.8, 28.0, 25.8, 23.9, 21.9, 19.2, 18.6, 16.4. 14.4, 14.1, 13.9, 12.6. IR (neat) v_{max}/cm^{-1} 3377 (w), 2967 (w), 2942 (m), 2873 (w), 1733 (s), 1727 (s), 1451 (m), 1361 (m), 1240 (s), 1156 (m), 1139 (s), 1010 (w), 978 (m), 900 (m). HRMS-ESI m/z calcd for $C_{33}H_{52}O_4$ $(M+Na)^+$: 535.3758, found 535.3759. **Step 2:** RuO_4 (101.4 mg, 0.61 mmol, 4.5 eq) and $NaIO_4$ (87.6 mg, 0.41 mmol, 3.00 eq) were added to a solution of the precursor (step 1, 70.0 mg, 0.14 mmol, 1.00 eq) in a 1:1 mixture of acetone/H_2O (2.0 ml). The reaction mixture was stirred for seven days at room temperature. After evaporation of the solvent *in vacuo*, the remaining residue was dissolved in DCM (20.0 ml) and the unsoluble RuO_4 was filtered off. The filtrate was washed once with 10 ml H_2O (10 ml) and sat. NaCl solution (10 ml). The organic layer was dried over Na_2SO_4, followed by removal of the solvent under reduced pressure. Purification of the crude product by preparative TLC (Cyh:EtOAc 4:1) afforded compound **16** (31.1 mg, 44%). HPLC (method C): t_R = 12.9 min, purity >95%. ^{1}H-NMR ($CDCl_3$, 400 MHz) δ: 4.46 (dd, 1H, J = 11.3 Hz, 5.0 Hz), 3.67 (s, 3H), 3.25 (td, 1H, J = 11.2, 4.5 Hz),2.28-2.22 (m,1H),), 2.17 (s, 3H), 2.13-2.07 (m, 1H), 2.03 (s, 3H), 2.02-1.96 (m, 1H), 1.91-1.86 (m, 1H), 1.60-1.25 (m, 15H), 1.19-1.15 (m, 1H), 1.09-1.05 (m, 2H), 0.98 (s, 3H), 0.97-0.93 (m, 1H), 0.92 (s, 3H), 0.84 (s, 6H), 0.82 (s, 3H), 0.80-0.77 (m, 1H). ^{13}C-NMR ($CDCl_3$, 101 MHz) δ: 212.3, 176.5, 171.0, 80.9, 56.4, 55.4, 51.4, 51.3, 50.3, 49.5, 42.2, 40.6, 38.4, 37.8, 37.4, 37.1, 36.6, 34.2, 31.5, 30.0, 29.7, 28.3, 27.9, 27.2, 23.7, 21.3, 20.9, 18.2, 16.5, 16.1, 15.9, 14.7. IR (neat) v_{max}/cm^{-1} 2944 (m), 2872 (w), 1733 (m), 1714 (s), 1455 (w), 1243 (s), 1152 (w), 1136 (m), 1013 (w), 978 (m). HRMS-ESI m/z calcd for $C_{32}H_{50}O_5$ $(M+Na)^+$: 537.3550, found 537.550.

(1R,3aS,5aR,5bR,7aR,9S,11aR,11bR,13aR,13bR)-N-(2-aminoethyl)-9-hydroxy-5a,5b,8,8,11a-pentamethyl-1-(prop-1-en-2-yl)icosahydro-3aH-cyclopenta[a]-chrysene-3a-carboxamide (17). **Step1:** To a solution of betulinic acid (100.0 mg, 0.22 mmol, 1.00 eq) and HATU (99.9 mg, 0.26 mmol, 1.20 eq) in DCM (10.0 ml) DIPEA (0.0076 ml, 0.48 mmol, 2.00 eq) and N-Boc-ethylenediamine (0.078 ml, 0.48 mmol, 2.20 eq) were added at 0 °C. The reaction mixture was stirred for 23 h at room temperature. The solution was washed once with H_2O (20.0 ml), sat. NaCl solution (20.0 ml) and H_2O (20.0 ml). The aqueous phase was extracted two times with DCM (20.0 ml). The combined organic layers were dried over Na_2SO_4, followed by solvent evaporation *in vacuo*. The crude product was purified by silica gel column chromatography (Cyh:EtOAc 3:1, silica gel: 23 g) to afford the pure intermediate (101.9 mg, 78%). HPLC (method B): t_R = 13.4 min, purity >95%. ^{1}H-NMR ($CDCl_3$, 400 MHz) δ: 7.22 (m, 1H), 6.28 (s, 1H), 4.72 (d, 1H, J = 2.4 Hz), 4.58 (d, 1H, J = 2.4 Hz), 3.36-3.32 (m, 1H), 3.30–3.32 (m, 2H), 3.21–3.06 (m, 2H), 2.44 (ddd, 1H, J = 12,8, 11.4 Hz, 3.6 Hz), 1.99-1.88 (m, 2H), 1.77-1.69 (m, 2H), 1.67 (s, 3H), 1.64 (t, 1H, J = 3.6, 3.6 Hz), 1.63-1.46 (m, 6H), 1.43 (s, 9H), 1.42 (s, 2H), 1.40-1.29 (m, 5H), 1.28-1.22 (m, 2H), 1.13 (dt, 1H, J = 13.1, 2.6, 2.6 Hz), 1.01-0.97 (m, 1H), 0.95 (s, 6H), 0.93 (s, 3H), 0.90–0.82 (m, 1H) 0.81 (s, 3H), 0.75 (s,

3H), 0.71–0.62 (m, 1H). ^{13}C-NMR ($CDCl_3$, 101 MHz) δ: 177.3, 157.0, 151.1, 109.4, 79.8, 79.1, 55.8, 50.8, 50.3, 46.9, 42.6, 40.9, 40.4, 39.0, 38.9, 38.5, 37.4, 34.6, 33.7, 31.0, 29.6, 28.5, 28.1, 27.6, 27.0, 25.8, 21.8, 19.6, 18.4, 16.3, 16.2, 15.5, 14.8. IR (neat) v_{max}/cm^{-1} 3363 (w), 3353 (w), 3332 (w), 2935 (s), 2866 (w), 1694 (m), 1638 (m), 1508 (s), 1450 (w), 1365 (m), 1319 (w), 1249 (m), 1167 (s), 1108 (w), 1043 (w), 1008 (w), 982 (w), 882 (m), 625 (w), 582 (w), 572 (w), 543 (m), 524 (w), 507 (w), 486 (w), 461 (w). HRMS-ESI m/z calcd for $C_{37}H_{62}N_2O_4$ $(M+Na)^+$: 621.4605, found 621.4602. **Step2:** The precursor (step1, 100.0 mg, 0.17 mmol, 1.00 eq) was dissolved in 4 M HCl/Dioxane (5.0 ml) and stirred for three days at room temperature. The solvent was removed under reduced pressure and the crude product was purified by silica gel column chromatography (DCM:MeOH 9:1, silica gel: 23 g) to afford the pure compound **17** (86.7 mg, 97%). HPLC (method B): t_R = 6.97 min, purity >95%. ^1H-NMR ($CDCl_3$, 400 MHz) δ: 8.07 (s, H), 7.90 (s, 1H), 4.65 (s, 1H), 4.54 (s, 1H), 3.31-3.23 (m, 2H), 3.04-2.92 (m, 2H), 2.84-2.74 (m, 2H), 2.24-2.11 (m, 1H), 1.88-1.79 (m, 1H), 1.78-1.66 (m, 1H), 1.63 (s, 3H), 1.59-1.53 (m, 1H), 1.48-1.38 (m, 5H), 1.38-1.19 (m, 9H), 1.18-095 (m, 4H), 0.95 (s, 3H), 0.86 (s, 3H), 0.84 (s, 3H), 0.83-0.78 (m, 1H), 0.76 (s, 3H), 0.65 (s, 3H), 0.62-0.57 (m, 1H). ^{13}C-NMR ($CDCl_3$, 101 MHz) δ: 176.3, 150.8, 109.2, 76.2, 54.9, 50.0, 49.6, 46.2, 41.9, 40.3, 38.6, 38.5, 38.3, 37.4, 36.7, 36.7, 36.4, 33.9, 32.2, 30.3, 28.9, 28.1, 27.1, 25.2, 20.5, 19.0,17.9, 15.9, 15.8, 15.8, 14.3. IR (neat) v_{max}/cm^{-1} 3395 (s), 3071 (w), 2937 (m), 2867 (w), 1638 (m), 1547 (w), 1468 (w), 1447 (w), 1391 (w), 1318 (w), 1248 (w), 1170 (w), 1075 (w), 1044 (w), 994 (s), 879 (m), 825 (w), 764 (w), 572 (w), 543 (w), 494 (w), 471 (w), 433 (w). HRMS-ESI m/z calcd for $C_{32}H_{54}N_2O_2$ $(M+H)^+$: 499.4258, found 499.4260.

*2-((((1R,3aS,5aR,5bR,7aR,9S,11aR,11bR,13aR,13bR)-3a-Carboxy-5a,5b,8,8,11a-pentamethyl-1-(prop-1-en-2-yl)-icosahydro-1H-cyclopenta[a]chrysen-9-yl)oxy)-carbonyl)-thiophene-3-carboxylic acid (**18**).* Compound **18** was obtained from betulinic acid (100.0 mg, 0.22 mmol, 1.00 eq) and 2,3-thiophene dicarboxylic anhydride (135.0 mg, 0.88 mmol, 4.00 eq) according to general procedure A. Reaction time: 5 days (100 °C). Purification: Preparative HPLC (H_2O:MeCN 5% → 95% MeCN, solid phase material: C18). Yield: 8.8 mg (7%). HPLC (method A): t_R = 11.0 min, purity >99%. ^1H-NMR (DMSO-d_6, 600 MHz) δ: 7.83 (d, 1H, J = 5.1 Hz 7.27 (d, 1H, J = 5.1 Hz),4.70-4.68 (m, 1H), 4.62-4.59 (m, 1H), 4.57-4.56 (m, 1H), 2.92 (m, 1H), 2.25-2.20 (m, 1H), 2.13-2.10 (m, 1H), 1.83-1.77 (m, 2H), 1.70-1.66 (m, 2H), 1.65 (s, 1H), 1.64-1.61 (m, 1H), 1.53 (td, 1H, J = 11.3 Hz), 1.49-1.45 (m, 1H), 1.42-1.36 (m, 6H), 1.34-1.30 (m, 3H), 1.24-1.21 (m, 1H), 1.19-1.15 (m, 1H), 1.03-0.98 (m, 2H), 0.96 (s, 3H), 0.90 (s, 3H), 0.88 (s, 3H) 0.87-0.85 (m, 1H), 0.82 (s, 3H), 0.79 (s, 3H). ^{13}C-NMR (DMSO-d_6, 151 MHz) δ: 177.3, 164.0, 162.1, 150.4, 136.9, 131.3, 128.3, 109.7, 81.8, 55.4, 54.8, 49.7, 48.6, 46.7, 42.1, 40.3, 37.7, 37.6, 37.6, 36.7, 36.4, 33.8, 31.7, 30.1, 29.2, 27.7, 25.1, 23.0, 20.5, 19.0, 17.7, 16.5, 15.7, 15.5, 14.4. IR (neat) v_{max}/cm^{-1} 3559(w), 2939 (m), 2868 (m), 1719 (m), 1683 (w), 1626 (w), 1446 (w), 1386 (w), 1259 (s), 982 (w), 887 (w), 749 (w). HRMS-ESI m/z calcd for $C_{36}H_{50}O_6S$ $(M+Na)^+$: 633.3220, found 633.3216.

*(1R,3aS,5aR,5bR,7aR,9S,11aR,11bR,13aR,13bR)-9-((2-Carboxybenzoyl)oxy)-5a,5b,8,8,11a-pentamethyl-1-(prop-1-en-2-yl)icosahydro-3aH-cyclopenta[a]-chrysene-3a-carboxylic acid (**19**).*[7] Compound **19** was obtained from betulinic acid (200.0 mg, 0.44 mmol, 1.0 eq) and phthalic anhydride (259.0 mg, 1.75 mmol, 4.00 eq) according to general procedure B. Reaction time: 120 minutes. Purification: Reversed phase chromatography (H_2O:MeCN 5% → 95% MeCN), solid phase material: C18, 32 g). Yield: 201.0 mg (76 %). HPLC (method B): t_R = 12.3 min, purity >99%. ^1H-NMR (DMSO-d_6, 600 MHz) δ: 12.60 (s, 2H), 7.72-7.69 (m, 1H), 7.63-7.59 (m, 3H), 4.70 (d, 1H, J = 2.5 Hz), 4.60 (dd, 1H, J = 11.0, 5.3 Hz), 4.57-4.56 (m, 1H), 2.97-2.93 (m, 1H), 2.26-2.21 (m, 1H), 2.13-2.11 (m, 1H), 1.84-0.99 (m, 27H), 0.96 (s, 3H), 0.89 (s, 6H), 0.83 (s, 3H), 0.81 (s, 3H). ^{13}C-NMR (DMSO-d_6, 151 MHz) δ: 177.2, 168.2, 167.1, 150.3, 132.5, 132.4, 131.0, 130.9, 128.6, 128.2, 109.6, 55.4, 54.8, 49.6, 48.5, 46.6, 42.0, 40.3, 40.0, 37.7, 37.6, 37.5, 36.6, 36.3, 33.7, 31.7, 30.1, 29.2, 28.9, 27.7, 22.7, 20.5, 25.0, 18.9, 17.7, 16.4, 15.8, 15.7, 14.4. IR (neat) v_{max}/cm^{-1} 3071 (w), 2944 (s), 2871 (w), 1698 (s), 1642 (w), 1450 (w), 1391 (w), 1377 (w), 1346 (w), 1286 (s), 1192 (w), 1129 (m), 1074 (w), 965 (w), 942 (w), 883 (w), 741 (w), 703 (w). HRMS-ESI m/z calcd for $C_{38}H_{51}O_6$ $(M-H)^-$: 603.3691, found 603.3691.

*(1R,3aS,5aR,5bR,7aR,11aR,11bR,13aR,13bR)-5a,5b,-8,8,11a-Pentamethyl-9-oxo-1-(prop-1-en-2-yl)icosa-hydro-3aH-cyclopenta[a]chrysene-3a-carboxylic acid (**20**).*[8] A mixture of H_2O (9.0 µl) and DCM (9.0 ml) was slowly added to a suspension of betulinic acid (200.0 mg, 0.44 mmol, 1.00 eq) and DMP (185.7 mg, 0.44 mmol, 1.00 eq) in DCM (1.0 ml) at room temperature. The solution was stirred for 26.5 h and diluted with diethylether (10.0 ml). The solution was washed once with a mixture of 10% $Na_2S_2O_4$ (5.0 ml) and sat. $NaHCO_3$ solution (15.0 ml), once with H_2O (15 ml), and once with sat. NaCl solution (15.0 ml). The organic layer was dried over Na_2SO_4 and the solvent was removed under reduced pressure to

afford the pure product **20** (188.9 mg, 92%). HPLC (method B): t_R = 12.3 min, purity >99%. ^{1}H-NMR ($CDCl_3$, 600 MHz) δ: 4.71 (s, 1H), 4.58 (s, 1H), 2.96 (td, 1H, J = 10.7, 5.1 Hz), 1.85-1.78 (m, 2H), 1.66 (s, 1H), 1.65-1.61 (m, 1H), 1.56-1.51 (m, 1H), 1.46-1.29 (m, 12H), 1.26-1.21 (m, 1H), 1.19 (t, 1H, J = 7.1 Hz), 1.17-1.09 (m, 1H), 1.00 (s, 3H), 0.96 (s, 3H), 0.94 (s, 3H), 0.92 (s, 3H), 0.86 (s, 3H), 0.85-0.77 (m, 1H). ^{13}C-NMR ($CDCl_3$, 151 MHz) δ: 177.2, 150.3, 109.6, 59.7, 55.4, 53.8, 49.0, 48.5, 46.6, 46.5, 42.0, 40.1, 38.8, 37.7, 36.3, 36.3, 33.6, 33.1, 30.1, 29.2, 26.4, 25.1, 21.0, 19.1, 18.9, 15.6, 15.4, 14.3. IR (neat) v_{max}/cm^{-1} 2932 (s), 2866 (w), 1703 (w), 1687 (s), 1458 (s), 1375 (w), 1261 (w), 1244 (w), 1177 (s), 1165 (w), 1112 (w), 1027 (s), 1002 (w), 941 (w), 887 (s), 800 (w), 763 (w). HRMS-ESI m/z calcd for $C_{30}H_{46}O_3$ $(M+Na)^+$: 477.3339, found 477.3339.

*Ethyl ((1R,3aS,5aR,5bR,7aR,9S,11aR,11bR,13bR)-9-hy-droxy-5a,5b,8,8,11a-pentamethyl-1-(prop-1-en-2-yl)icosa-hydro-1H-cyclopenta[a]chrysene-3a-car-bonyl)glycinate (**21**).*[9] DIPEA (1.91 ml, 10.95 mmol, 10.00 eq) was added at 0 °C to a solution of betulinic acid (500.0 mg, 1.10 mmol, 1.00 eq), glycine ethyl ester hydrochloride (458.4 mg, 3.28 mmol, 3.00 eq), and HATU (1040.7 mg, 2.74 mmol, 1.50 eq) in DCM (12.0 ml). The mixture was stirred for five days at room temperature. The solvent was removed under reduced pressure and the residue was dissolved in EtOAc (20.0 ml). The organic layer was washed once with H_2O (10.0 ml), sat. NaCl solution (10.0 ml), sat. $NaHCO_3$ solution (10.0 ml), sat. NaCl solution (10.0 ml) and H_2O (10.0 ml). The organic layer was dried over Na_2SO_4 and the solvent was evaporated under reduced pressure. The crude product was purified by silica gel column chromatography (Cyh:EtOAc 19:1, silica gel: 37 g) to give pure compound **21** (485.4 mg, 82%). HPLC (method B): t_R = 11.9 min, purity >99%. ^{1}H-NMR ($CDCl_3$, 600 MHz) δ: 6.06 (t, 1H, J = 5.2 Hz), 4.73 (d, 1H, J = 2.4 Hz), 4.59 (d, 1H, J = 2.4 Hz), 4.21 (q, 2H, J = 7.1 Hz), 4.00 (dd, 2H, J = 5.25, 3.66 Hz), 3.18 (dd, 1H, J = 11.5, 4.7 Hz), 3.09 (td, 1H, J = 11.1, 4,5 Hz), 2.43 (td, 1H, J = 11.5, 3.7 Hz), 2.02-1.92 (m, 2H), 1.91-1.79 (m, 1H), 1.72-1.69 (m, 1H), 1.68 (s, 3H), 1.66-1.64 (m, 1H), 1.63-1.57 (m, 3H), 1.57-1.55 (m, 1H), 1.55-1.53 (m, 1H), 1.52-1.45 (m, 2H), 1.42-1.35 (m, 5H), 1.29 (t, 2H, J = 7.1 Hz), 1.26-1.24 (m, 2H), 1.19-1.13 (m, 1H), 1.03-0.97 (m, 1H), 0.97 (s, 3H), 0.96 (s, 3H), 0.92 (s, 3H), 0.89-0.86 (m, 1H), 0.81 (s, 3H), 0.75 (s, 3H), 0.69-0.65 (m, 1H). ^{13}C-NMR ($CDCl_3$, 151 MHz) δ: 176.8, 170.6, 151.0, 109.5, 79.2, 61.4, 55.7, 55.4, 50.6, 50.0, 46.6, 42.5, 41.2, 40.7, 38.8, 38.7, 38.2, 37.7, 37.2, 34.3, 33.6, 30.8, 29.4, 27.9, 27.4, 25.6, 20.9, 19.4, 16.0, 14.6, 14.3. IR (neat) v_{max}/cm^{-1} 2936 (w), 2869 (w), 1696 (m), 1520 (w), 1456 (w), 1191 (w), 1159 (w), 1083 (s), 1029 (w), 789 (m), 746 (w), 470 (s). HRMS-ESI m/z calcd for $C_{34}H_{55}NO_4$ $(M+Na)^+$: 564.4023, found 564.4028.

*(1R,3aS,5aR,5bR,7aR,9S,11aR,11bR,13aR,13bR)-9-((3-Carboxy-2-naphthoyl)oxy)-5a,5b,8,8,11a-penta-methyl-1-(prop-1-en-2-yl)icosahydro-3aH-cyclopenta-[a]chrysene-3a-carboxylic acid (**22**).* Product **22** was obtained from betulinic acid (50.0 mg, 0.11 mmol, 1.00 eq) and 2,3-naphthalic anhydride (86.8 mg, 0.44 mmol, 4.00 eq) according to general procedure B. Reaction time: 70 minutes. Purification: Reversed phase chromatography (H_2O (5 mM NH_4OAc): MeCN 0% → 95% MeCN), solid phase material: C18, 20 g). Yield: 41.0 mg (57 %). HPLC (method A): t_R = 11.5 min, purity >95%. ^{1}H-NMR (DMSO-d_6, 600 MHz) δ: 9.89 (s, 2H), 8.42 (s, 1H), 8.37 (s, 1H,), 8.29 (s, 1H), 8.25 (s, 1H), 8.15-8.11 (m, 2H), 7.73-7.70 (m, 2H), 4.71 (d, 1H, J = 2.0 Hz), 4.70 (d, 1H, J = 2.0 Hz), 4.67 (dd, 1H, J = 11.5, 4.8 Hz), 4.59-4.58 (m, 1H), 4.57-4.56 (m, 1H), 4.26-4.25 (m, 1H), 2.99-2.95 (m, 1H), 2.28-2.23 (m, 1H), 2.15-2.11 (m, 1H), 1.84-0.99 (m, 27H), 0.98-0.66 (m, 15H). ^{13}C-NMR (DMSO-d_6, 151 MHz) δ: 177.2, 168.2, 168.1, 168.0, 167.2, 150.3, 132.9, 132.8, 132.7, 132.7, 129.8, 129.5, 129.1, 129.0, 128.8, 128.7, 128.6, 128.6, 128.5, 128.4, 109.6, 81.7, 76.7, 55.4, 54.9, 54.8, 52.4, 49.9, 49.7, 48.5, 46.6, 42.0, 42.0, 40.2, 38.4, 37.8, 37.6, 36.7, 36.6, 36.3, 33.7, 31.7, 30.1, 29.2, 28.0, 27.8, 26.3, 25.0, 22.8, 20.5, 20.4, 18.9, 17.7, 16.5, 16.5, 15.9, 15.8, 15.7, 15.7, 14.3, 14.3. IR (neat) v_{max}/cm^{-1} 2942 (s), 2870 (w), 1689 (s), 1643 (w), 1605 (w), 1449 (w), 1391 (w), 1376 (w), 1318 (w), 1287 (w), 1251 (w), 1181 (s), 1125 (w), 1032 (w), 962 (w), 943 (w), 883 (m), 789 (w), 765 (m), 749 (w), 476 (w). HRMS-ESI m/z calcd for $C_{42}H_{53}O_6$ $(M-H)^-$: 653.3848, found 653.3853.

*3-((((1R,3aS,5aR,5bR,7aR,9S,11aR,11bR,13aR,13bR)-3a-Carboxy-5a,5b,8,8,11a-pentamethyl-1-(prop-1-en-2-yl)-icosahydro-1H-cyclopenta[a]chrysen-9-yl)oxy)-carbonyl)-pyrazine-2-carboxylic acid (**23**).* Product **23** was obtained from betulinic acid (50.0 mg, 0.11 mmol, 1.00 eq) and pyrazine-2,3-dicarboxylic anhydride (65.7 mg, 0.44 mmol, 4.00 eq) according to general procedure A. Reaction time: 18.0 h (rt). Purification: Reversed phase (C18) chromatography. Yield: 39.7 mg (60%). HPLC (method A): t_R = 7.9 min, purity >99%. ^{1}H-NMR (CD_3OD, 400 MHz) δ: 8.78 (s, 2H), 4.79 (dd, 1H, J = 11.8, 4.9 Hz), 4.72 (d, 1H, J = 1.8 Hz), 4.51-4.49 (m, 1H), 3.06-3.00 (m, 1H), 2.36-2.23 (m, 2H), 1.93-1.08 (m, 25H), 1.04 (s, 3H), 0.99 (s, 3H), 0.98 (s, 3H), 0.92 (s, 3H), 0.88 (s, 3H). ^{13}C-NMR (CD_3OD, 101 MHz) δ: 180.0, 167.6, 166.5,

152.0, 147.5, 146.8, 146.5, 110.2, 85.3, 57.5, 57.0, 51.9, 50.5, 48.5, 43.6, 42.0, 39.7, 39.2, 38.3, 38.1, 35.5, 33.3, 31.7, 30.9, 28.5, 26.9, 24.2, 22.2, 19.6, 19.3, 17.0, 16.8, 16.7, 16.7,15.2. IR (neat) v_{max}/cm^{-1} 3345 (w), 3070 (w), 2950 (s), 2874 (w), 1692 (m), 1598 (s), 1452 (w), 1374 (s), 1297 (m), 1177 (w), 1158 (m), 1135 (w), 1095 (m), 1015 (w), 946 (w), 884 (w). HRMS-ESI m/z calcd for $C_{36}H_{49}N_2O_6$ $(M-H)^-$: 605.3596, found: 605.3597.

*(1R,3aS,5aR,5bR,7aR,9S,11aR,11bR,13aR,13bR)-9-((2-Carboxy-3,6-difluoro-benzoyl)oxy)-5a,5b,8,8,11a-penta-methyl-1-(prop-1-en-2-yl)icosahydro-3aH-cyclopenta[a]-chrysene-3a-carboxylic acid (**24**).* Product **24** was obtained from betulinic acid (50.0 mg, 0.11 mmol, 1.00 eq) and 3,6-difluorophthalic anhydride (80.6 mg, 0.44 mmol, 4.00 eq) according to general procedure B. Reaction time: 90.0 minutes. Purification: Reversed phase (C18) chromatography (H_2O (5 mM NH_4OAc): MeCN 0% → 95% MeCN), solid phase material: 20 g). Yield: 40.0 mg (57%). HPLC (method A): t_R = 9.2 min, purity >99%. ^1H-NMR (CD_3OD, 400 MHz) δ: 7.25-7.19 (m, 1H), 7.14-7.08 (m, 1H), 4.74-4.71 (m, 2H), 4.63-4.62 (m, 1H), 3.08-3.02 (m, 1H), 2.36-2.23 (m, 2H), 1.97-1.09 (m, 27H), 1.06 (s, 3H), 1.01 (s, 3H), 1.00 (s, 3H), 0.94 (s, 3H), 0.92 (s, 3H). ^{13}C-NMR (CD_3OD, 101 MHz) δ: 180.0, 152.0, 110.2, 84.5, 57.5, 57.1, 51.9, 50.5, 43.6, 42.0, 39.7, 39.0, 38.3, 38.1, 35.5, 33.4, 31.7, 30.8, 28.5, 26.9, 24.3, 22.1, 19.6, 19.3, 17.1, 16.7, 15.1. IR (neat) v_{max}/cm^{-1} 2929 (s), 2869 (w), 1713 (w), 1686 (s), 1642 (w), 1475 (m), 1451 (w), 1389 (w), 1376 (w), 1349 (w), 1274 (w), 1239 (s), 1194 (w), 1131 (w), 1107 (w), 1043 (w), 985 (w), 962 (w), 943 (w), 882 (w), 823 (m), 726 (m). HRMS-ESI m/z calcd for $C_{38}H_{49}F_2O_6$ $(M-H)^-$: 639.3503 found, 639.3505.

*(1R,3aS,5aR,5bR,7aR,9S,11aR,11bR,13aR,13bR)-9-((2-Carboxy-4,5-difluorobenzoyl)oxy)-5a,5b,8,8,11a-penta-methyl-1-(prop-1-en-2-yl)icosahydro-3aH-cyclopenta[a]-chrysene-3a-carboxylic acid (**25**).* Product **25** was obtained from betulinic acid (50.0 mg, 0.11 mmol, 1.00 eq) and 4,5-difluorophthalic anhydride (80.6 mg, 0.44 mmol, 4.00 eq) according to general procedure A. Reaction time: 18 h (rt). Purification: reversed phase preparative HPLC (MeCN:H_2O 5% → 95% MeCN, solid phase material: C18, 20 g). Yield: 32.0 mg (46%). HPLC (method A): t_R = 10.0 min, purity >99%. ^1H-NMR (CD_3OD, 400 MHz) δ: 7.60-7.55 (m, 1H), 4.74 (d, 1H, J = 1.9 Hz), 4.72 (dd, 1H, J = 11.0, 5.1 Hz), 4.63-4.62 (m, 1H), 3.07-3.03 (m, 1H), 2.36-2.25 (m, 2H), 2.00-1.07 (m, 27H), 1.05 (s, 3H), 1.01 (s, 3H), 0.97 (s, 3H), 0.94 (s, 3H). ^{13}C-NMR (CD_3OD, 101 MHz) δ: 180.0, 170.8, 167.3, 151.5, 151.9, 151.5, 135.8, 135.7, 130.1, 119.8, 118.8, 110.2, 84.5, 57.5, 57.5, 50.4, 51.9, 48.5, 43.6, 42.0, 39.6, 39.1, 38.3, 38.1, 35.5, 33.3, 31.7, 30.8, 28.7, 26.8, 24.2. 22.2, 19.6, 19.3, 17.2, 16.8, 16.7, 15.2. IR (neat) v_{max}/cm^{-1} 3067 (w), 2942 (m), 2870 (w), 1687 (s), 1643 (w), 1605 (w), 1516 (w), 1449 (w), 1375 (w), 1318 (m), 1248 (m), 1180 (s), 1116 (w), 1030 (w), 961 (w), 940 (w), 882 (m), 765 (m), 650 (w), 610 (w). HRMS-ESI m/z calcd for $C_{38}H_{49}F_2O_6$ $(M-H)^-$: 639.3503 found, 639.3500.

*(1R,3aS,5aR,5bR,7aR,9S,11aR,11bR,13aR,13bR)-9-((2-Carboxy-4,5-dimethoxybenzoyl)oxy)-5a,5b,8,8,-11a-penta-methyl-1-(prop-1-en-2-yl) icosahydro-3aH-cyclopenta[a]-chrysene-3a-carboxylic acid (**26**).* Product **26** was obtained from betulinic acid (50.0 mg, 0.11 mmol, 1.00 eq) and 5,6-dimethoxyisobenzofuran-1,3-dione (80.6 mg, 0.44 mmol, 4.00 eq) according to general procedure B. Reaction time: 120 minutes. Purification: Reversed phase chromatography (H_2O (5 mM NH_4OAc): MeCN 0% → 95% MeCN), solid phase material: C18, 20 g). Yield: 48.2 mg (66%). HPLC (method A): t_R = 9.7 min, purity >95%. ^1H-NMR ($CDCl_3$, 600 MHz) δ: 9.89 (s, 2H), 7.44 (s, 1H), 7.26 (s, 1H), 4.74 (s, 1H), 4.72 (dd, 1H, J = 11.6, 4.6 Hz), 4.61 (s, 1H), 3.96 (s, 3H), 3.95 (s, 3H), 3.02-2.97 (m, 1H), 2.29-1.02 (m, 27H), 0.99 (s, 3H), 0.95 (s, 3H), 0.93 (s, 3H) 0.89 (s, 3H), 0.88 (s, 3H). ^{13}C-NMR ($CDCl_3$, 151 MHz) δ: 183.0, 171.4, 168.2, 151.5, 150.8, 150.4, 126.5, 124.1, 113.2, 112.1, 109.9, 80.6, 56.6, 56.4, 55.6, 50.4, 49.4, 47.0, 42.6, 40.8, 38.5, 38.4, 38.2, 37.2, 34.3, 32.3, 30.7, 29.8, 28.3, 25.5, 23.1, 21.1, 19.5, 18.3, 16.8, 16.5, 16.3, 14.8. IR (neat) v_{max}/cm^{-1} 2953 (m), 2872 (w), 1688 (s), 1643 (w), 1605 (w), 1573 (w), 1518 (w), 1445 (w), 1422 (w), 1403 (w), 1361 (m), 1284 (m), 1258 (w), 1209 (s), 1175 (m), 1138 (w), 1107 (w), 1058 (w), 1025 (w), 980 (w), 963 (w), 930 (w), 914 (w), 880 (m), 795 (w), 763 (w), 732 (w), 646 (w). HRMS-ESI m/z calcd for $C_{40}H_{56}O_8Na$ $(M+Na)^+$: 687.3867found.: 687.3867.

*(R)-2-((1R,3aS,5aR,5bR,7aR,9S,11aR,11bR,13aR,13b-R)-9-Acetoxy-3a-(methoxycarbonyl)-5a,5b,8,8,-11a-pentamethyl-icosahydro-1H-cyclopenta[a]chry-sen-1-yl)propanoic acid (**27**).* $KMnO_4$ (616.4 mg, 3.90 mmol, 20.00 eq) and $CuSO_4$ x 5 H_2O (486.8 mg, 1.95 mmol, 10.00 eq) were added under an argon atmosphere to a solution of compound **16** (step 1), (100.0 mg, 0.195 mmol, 1.00 eq) in a mixture of DCM (10.0 ml), tert-butanol (0.5 ml) and H_2O (0.15 ml). The reaction mixture was stirred for 5 days at room temperature. Then, the solution was diluted with DCM (10 ml) and washed three

times with sat. $NaHCO_3$ solution (10 ml), H_2O (10 ml) and sat NaCl solution (10 ml). The organic layer was dried over Na_2SO_4 followed by solvent evaporation under reduced pressure. The crude product was purified by preparative TLC (Cyh:EtOAc 1:1) to give pure product **27** (13.1 mg, 12%). HPLC (method C): t_R = 9.3 min, purity >99%. ^{1}H-NMR ($CDCl_3$, 400 MHz) δ: 4.48 (dd, 1H, J = 11.2, 5.2 Hz), 3.65 (s, 3H), 2.74 (dd, 1H, J = 7.1, 3.1 Hz),2.23-2.19), 2.19-2.14 (m, 1H), 2.04 (s, 3H), 1.83-1.73 (m, 2H), 1.71-1.66 (m, 3H), 1.64-1.60 (m, 2H), 1.52-1.46 (m, 2H), 1.40-1.31 (m, 9H), 1.28-1.26 (m, 1H), 1.16 (s, 3H), 1.14-1.10 (m, 1H), 1.03-0.96 (m, 1H), 0.99 (s, 3H), 0.90 (s, 3H), 0.85 (s, 3H), 0.84 (s, 3H), 0.83 (s, 3H), 0.81-0.77 (m, 1H). ^{13}C-NMR ($CDCl_3$, 101 MHz) δ: 181.1, 177.4, 171.1, 81.3, 57.2, 55.8, 51.6, 50.5, 50.1, 43.5, 42.9, 42.6, 41.1, 38.8, 38.5, 38.2, 37.5, 36.8, 34.7, 32.3, 30.1, 28.2, 27.4, 24.9, 24.1, 21.7, 21.3, 18.5, 17.6, 16.9, 16.5, 16.3, 14.9. IR (neat) v_{max}/cm^{-1} 2941 (s), 2871 (w), 1707 (s), 1454 (m), 1366 (m), 1269 (s), 1156 (w), 1135 (w), 1026 (m), 978 (m), 899 (w). HRMS-ESI m/z calcd for $C_{33}H_{52}O_6$ $(M+Na)^+$: 567.3656, found 567.3656.

*((1R,3aS,5aR,5bR,7aR,9S,11aR,11bR,13aR,13bR)-9-Hydroxy-5a,5b,8,8,11a-pentamethyl-1-(prop-1-en-2-yl)-icosahydro-3aH-cyclopenta[a]chrysen-3a-yl)me-thyl acetate (**29**).*[10] NEt_3 (0.24 ml, 1.75 mmol, 8.00 eq) and Ac_2O (22.7 µl, 0.24 mmol, 1.10 eq) were added to a suspension of betulin (100.0 mg, 0.21 mmol, 1.00 eq) and DMAP (3.0 mg, 0.02 mmol, 0.10 eq) in DCM (4.0 ml). After stirring for two days at room temperature, the solution was diluted with DCM (10.0 ml) and washed three times with H_2O (5.0 ml), sat. $NaHCO_3$ solution (5.0 ml), and sat. NaCl solution (5.0 ml). The organic layer was dried over Na_2SO_4 followed by solvent evaporation under reduced pressure. The crude product was purified by silica gel chromatography (Cyh:EtOAc 19:1, silica gel: 23 g) to give pure product **29** (52.5 mg, 50%). HPLC (method B): t_R = 13.7 min, purity >99%. ^{1}H-NMR ($CDCl_3$, 600 MHz) δ: 4.70-4.67 (m, 1H), 4.60-4.56 (m, 1H), 4.24 (d, 1H, J = 11.1 Hz), 3.88-3.83 (m, 1H), 3.20-3.15 (m, 1H), 2.47-2.40 (m, 1H), 2.07), 1.99-1.92 (m, 1H), 1.86-1.82 (m, 1H), 1.79-1.74 (m, 1H), 1.71-1.69 (m, 1H), 1.68 (s, 1H), 1.67-1.62 (m, 3H), 1.61-1.57 (m, 2H), 1.55 (m, 2H), 1.53-1.50 (m, 1H), 1.43-1.37 (m, 5H), 1.28-1.20 (m, 3H), 1.14-1.03 (m, 3H), 1.03 (s, 3H), 0.97 (s, 3H), 0.96 (s, 3H), 0.92-0.86 (m, 1H), 0.82 (s, 3H), 0.76 (s, 3H), 0.69-0.66 (m, 1H). ^{13}C-NMR ($CDCl_3$, 151 MHz) δ: 171.9, 150.5, 110.2, 79.3, 63.1, 55.6, 50.7, 49.2, 48.1, 46.7, 43.0, 41.2, 39.2, 39.1, 38.0, 37.5, 34.9, 34.5, 30.2, 29.9, 28.3, 27.8, 27.4, 25.5, 21.3, 21.1, 19.4, 18.6, 16.5, 16.4, 15.7, 15.1. IR (neat) v_{max}/cm^{-1} 3560 (m), 2964 (w), 2939 (m), 2868 (w), 1719 (s), 1452 (m), 1387 (m), 1367 (m), 1257 (s), 1043 (s), 983 (w), 888 (m). HRMS-ESI m/z calcd for $C_{32}H_{52}O_3$ $(M+Na)^+$: 507.3809, found 507.3808.

*(Z)-4-(((1R,3aS,5aR,5bR,7aR,9S,11aR,11bR,13aR,-13bR)-9-Hydroxy-5a,5b,8,8,11a-pentamethyl-1-(prop-1-en-2-yl)-icosahydro-3aH-cyclopenta[a]chrysen-3a-yl)methoxy)-4-oxobut-2-enoic acid (**30**).*[11] A mixture consisting of betulin (50.0 mg, 0.11 mmol, 1.00 eq), maleic anhydride (88.6 mg, 0.90 mmol, 8.00 eq), and DMAP (1.4 mg, 0.011 mmol, 0.10 eq) in pyridine (1.5 ml) was stirred for 17 h at room temperature. The solution was then diluted with EtOAc (20.0 ml) and washed with 1 M HCl (2x10.0 ml). The organic layer was dried over Na_2SO_4 and the solvent was removed under reduced pressure. Silica gel chromatographic purification (Cyh:EtOAc 9:1, silica gel: 14 g) afforded pure product **30** (8.7 mg, 14%). HPLC (method B): t_R = 11.9 min, purity >99%. ^{1}H-NMR ($CDCl_3$, 400 MHz) δ: 6.35 (m, 2H), 4.71 (s, 1H), 4.56 (s, 1H), 4.32 (d, 1H, J = 11.1 Hz), 3.82 (d, 1H, J = 11.1 Hz), 3.01-2.92 (m, 1H), 2.47-2.41 (m, 1H), 1.96-1.94 (m, 1H), 1.80-1.70 (m, 2H), 1.65 (s, 3H), 1.64-1.50 (m, 5H), 1.49-1.39 (m, 3H), 1.39-1.29 (m, 5H), 1.26-1.18 (m, 3H), 1.12-1.01 (m, 2H), 0.99 (s, 3H), 0.98-0.96 (m, 1H), 0.94 (s, 3H), 0.87 (s, 3H), 0.86-0.81 (m, 1H), 0.77 (s, 3H), 0.66 (s, 3H), 0.64-0.61 (m, 1H). ^{13}C-NMR ($CDCl_3$, 101 MHz) δ: 166.5, 165.7, 149.8, 131.7, 128.2, 109.9, 76.7, 62.5, 54.8, 49.7, 48.3, 46.9, 46.0, 40.1, 40.3, 38.2, 37.1, 36.7, 34.0, 33.7, 29.1, 29.0, 28.1, 27.1, 26.6, 24.7, 20.3, 18.8, 17.9, 15.9, 15.8, 15.6, 14.5. IR (neat) v_{max}/cm^{-1} 3394 (m), 2933 (w), 2870 (w), 1714 (m), 1641 (w), 1024 (w), 993 (s), 892 (w), 824 (m), 763 (w), 524 (w), 515 (w). HRMS-ESI m/z calcd $C_{34}H_{52}O_5$ $(M+Na)^+$: 563.3707, found 563.3707.

*4-(((1R,3aS,5aR,5bR,7aR,9S,11aR,11bR,13aR,13bR)-9-Hydroxy-5a,5b,8,8,11a-pentamethyl-1-(prop-1-en-2-yl)-icosahydro-3aH-cyclopenta[a]chrysen-3a-yl)me-thoxy)-4-oxobutanoic acid (**31**).*[12] A solution of betulin (500.0 mg, 1.11 mmol, 1.00 eq), succinic anhydride (169.5 mg, 1.69 mmol, 8.00 eq), and DMAP (13.8 mg, 0.11 mmol, 0.10 eq) in pyridine (5.0 ml) was stirred for 16 h at room temperature. After completion of the reaction, the solution was diluted with EtOAc (20.0 ml), washed with 1 M HCl (2x 10.0 ml) and dried over Na_2SO_4. After removal of the solvent under reduced pressure, the crude product was purified by silica gel chromatography (Cyh:EtOAc 3:1, silica gel: 32 g) to give product **31** (551.1 mg, 90%). HPLC (method B): t_R = 12.9 min, purity >95%. ^{1}H-NMR ($CDCl_3$, 600 MHz) δ: 4.70 (s, 1H), 4.56 (s, 1H), 4.29-4.22 (m, 2H), 3.77 (d, 1H, J = 11.1 Hz), 2.99-2.94 (m, 1H), 2.54-2.51 (m, 1H), 2.49-2.42 (m, 2H), 1.94-1.84 (m, 1H), 1.77-1.68 (m, 2H),

1.65 (s, 1H), 1.63-1.59 (m, 3H), 1.58-1.52 (m, 2H), 1.48-1.39 (m, 3H), 1.39-1.27 (m, 5H), 1.27-1.13 (m, 3H), 1.10-0.99 (m, 2H), 0.98 (s, 3H), 0.97-0.95 (m, 1H), 0.94 (s, 3H), 0.87 (s, 3H), 0.86-0.79 (m, 1H), 0.77 (s, 3H), 0.66 (s, 3H), 0.65-0.59 (m, 1H). ^{13}C-NMR ($CDCl_3$, 151 MHz) δ: 173.4, 172.2, 149.7, 109.8, 76.8, 61.5, 54.6, 49.7, 48.2, 47.0, 46.1, 42.2, 40.3, 38.5, 38.2, 37.0, 36.6, 34.0, 29.2, 29.0, 28.9, 28.7, 28.0, 27.2, 26.6, 24.9, 20.2, 18.8, 17.9, 15.9, 15.7, 15.6, 14.5. IR (neat) v_{max}/cm^{-1} 3324 (w), 2939 (s), 2866 (w), 2586 (w), 1731 (m), 1707 (s), 1388 (m), 1259 (m), 1160 (m), 1014 (s), 525 (w). HRMS-ESI m/z calcd for $C_{32}H_{50}O_4$ $(M+Na)^+$: 521.3601, found 521.3600.

4-(((1R,3aS,5aR,5bR,7aR,9S,11aR,11bR,13aR,13bR)-9-((3-Carboxypropanoyl)oxy)-5a,5b,8,8,11a-penta-methyl-1-(prop-1-en-2-yl)icosahydro-3aH-cyclopenta-[a]chrysen-3a-yl)-methoxy)-4-oxobutanoic acid (32).[13] Product **32** was obtained as a by-product during the synthesis of compound 31. Yield: 73.0 mg (10%). HPLC (method B): t_R = 11,7 min, purity >95%. ^{1}H-NMR (DMSO-d_6, 600 MHz) δ: 4.70 (s, 1H), 4.56 (s, 1H), 4.41-4.34 (m, 1H), 4.21 (d, 1H, J = 11.1 Hz), 3.77 (d, 1H, J = 11.0 Hz), 2.50-2.40 (m, 19H), 1.97-1.83 (m, 1H), 1.75-1.68 (m, 2H), 1.65 (s, 1H), 1.64-1.40 (m, 9H), 1.39-1.26 (m, 6H), 1.23-1.17 (m, 1H), 1.09-1.03 (m, 1H), 0.99 (s, 3H), 0.95 (s, 3H), 0.94-0.88 (m, 1H), 0.81 (s, 3H), 0.80 (s, 3H), 0.79 (s, 4H). ^{13}C-NMR (DMSO-d_6, 151 MHz) δ: 173.3, 172.2, 171.6, 149.8, 109.9, 80.0, 61.7, 54.6, 49.5, 48.2, 47.0, 46.1, 42.2, 40.9, 37.7, 37.4, 37.0, 36.6, 34.0, 33.6, 29.2, 29.2, 29.0, 28.9, 28.8, 28.8, 27.6, 26.6, 24.7, 23.3, 20.3, 18.8, 17.7, 16.4, 15.8, 15.6, 14.5. IR (neat) v_{max}/cm^{-1} 2927 (m), 2871 (w), 1720 (s), 1456 (w), 1376 (w), 1254 (w), 1172 (m), 967 (m), 882 (w), 731 (w). HRMS-ESI m/z calcd for $C_{38}H_{58}O_8$ $(M+Na)^+$: 665.4024, found 665.4023.

*((((1R,3aS,5aR,5bR,7aR,9S,11aR,11bR,13aR,13bR)-3a-(((3-Carboxypropanoyl)oxy)methyl)-5a,5b,8,8,11a-penta-methyl-1-(prop-1-en-2-yl)icosahydro-1H-cyclo-penta[a]-chrysen-9-yl)oxy)carbonyl)-3,6-difluoro-benzoic acid (**33**).* Product **33** was obtained from educt **31** (100.0 mg, 0.18 mmol, 1.00 eq) and 3,6-difluorophthalic anhydride (67.8 mg, 0.37 mmol, 2.00 eq) according to general procedure A. Reaction time: 4 days (100 °C). Purification: preparative HPLC (H_2O:MeCN 5% → 95% MeCN, solid phase material: C18). Yield: 45.0 mg (34%). HPLC (method C): t_R = 8.1 min, purity >95%. ^{1}H-NMR: (DMSO-d_6, 600 MHz) δ: 7.55 (2x d, 2H, J = 6.4 Hz), 4.71 (d, 1H, J = 2.6 Hz),4.65 (t, 1H, J = 8.1 Hz), 4.52 (s, 1H), 4.27 (d, 1H, J = 11.1 Hz), 3.77 (d, 1H, J = 11.1 Hz), 2.54-2.52 (m, 1H), 2.49-2.42), 1.94-1.86 (m, 1H), 1.78-1.68 (m, 3H), 1.67-1.60 (m, 8H), 1.59-1.53 (m, 1H), 1.51-1.46 (m, 1H), 1.42-1.29 (m, 6H), 1.24-1.20 (m, 2H), 0.90 (s, 3H), 0.89-0.85 (m, 1H), 0.82 (s, 3H), 0.81 (s, 3H). ^{13}H-NMR (DMSO-d_6, 151 MHz) δ: 173.4, 172.3, 163.2, 157.4, 145.4, 82.9, 61.7, 54.4, 49.4, 48.2, 47.0, 46.1, 42.3, 40.4, 37.6, 37.5, 37.5, 36.6, 34.0, 33.5, 29.2, 29.0, 28.9, 28.7, 27.5, 26.6, 24.7, 22.8, 20.3, 18.7, 17.7, 16.4, 15.6, 14.5. IR (neat) v_{max}/cm^{-1} 2944 (m), 2872 (w), 1710 (s), 1474 (m), 1246 (s), 1157 (m), 985 (m), 963 (m), 822 (w), 726 (w). HRMS-ESI m/z calcd for $C_{42}H_{56}O_8F_2$ $(M+Na)^+$: 749.3835, found 749.3832.

*2-((((1R,3aS,5aR,5bR,7aR,9S,11aR,11bR,13aR,13bR)-3a-(((3-Carboxypropanoyl)oxy)methyl)-5a,5b,8,8,11a-penta-methyl-1-(prop-1-en-2-yl)icosahydro-1H-cyclo-penta[a]-chrysen-9-yl)oxy)carbonyl)-4,5-difluoro-benzoic acid (**34**).* Product **34** was obtained from starting material 31 (100.0 mg, 0.18 mmol, 1.00 eq) and 4,5-difluorophthalic anhydride (67.8 mg, 0.37 mmol, 2.00 eq) according to general procedure A. Reaction time: 4 days (100 °C). Purification: preparative HPLC (H_2O:MeCN 5% → 95% MeCN, solid phase material: C18). Yield: 56.70 mg (42%). HPLC (method C): t_R = 8.8 min, purity >99%. ^{1}H-NMR (DMSO-d_6, 600 MHz) δ: 7.83-7.78 (m, 1H), 4.71 (s, 1H),4.60 (dd, 1H, J = 10.6, 5.6 Hz), 4.57 (s, 1H), 4.27 (d, 1H, J = 11.1 Hz), 3.77 (d, 1H, J = 11.1 Hz), 2.54-2.51 (m, 2H), 2.49-2.43), 1.94-1.86 (m, 1H), 1.77-1.68 (m, 3H), 1.68-1.60 (m, 8H), 1.60-1.53 (m, 1H), 1.51-1.44 (m, 1H), 1.44-1.30 (m, 6H), 1.24-1.18 (m, 2H), 0.89 (s, 3H), 0.88-0.84 (m, 1H), 0.83 (s, 3H), 0.81 (s, 3H) ^{13}H-NMR (DMSO-d_6, 151 MHz) δ: 173.3, 172.3, 166.2, 149.8, 82.5, 61.7, 54.7, 49.5, 48.2, 47.0, 46.1, 42.2, 40.4, 37.7, 37.6, 37.0, 36.6, 34.0, 33.5, 29.2, 29.0, 28.9, 28.7, 27.7, 26.6, 24.7, 22.7, 20.3, 18.8, 17.7, 16.4, 15.7, 15.6, 14.5. IR (neat) v_{max}/cm^{-1} 2942 (m), 2871 (w), 1708 (s), 1606 (w), 1319 (m), 1251 (m), 1178 (s), 1116 (m), 963 (w),882 (w), 764 (w). HRMS-ESI m/z calcd $C_{42}H_{56}O_8F_2$ $(M+Na)^+$: 749.3835, found 749.3839.

(1S,2R,4aS,6aS,6bR,8aR,10S,12aR,12bR,14bR)-10-((2-Carboxy-3,6-difluorobenzoyl)oxy)-1,2,6a,6b,9,9,-12a-heptamethyl-1,3,4,5,6,6a,6b,7,8,8a,9,10,11,12,-12a,12b,-13,14b-octadecahydropicene-4a(2H)-car-boxylic acid (36). Product **36** was obtained from ursolic acid (100.0 mg, 0.22 mmol, 1.00 eq) and 3,6-difluorophthalic anhydride (80.6 mg, 0.44 mmol, 2.00 eq) according to general procedure A. Reaction time: 9 days (100 °C). Purification: preparative HPLC (H_2O:MeCN 5% → 95% MeCN, solid phase material: C18). Yield: 19.2 mg (14%). HPLC (method A): t_R = 9.3 min, purity >99%. ^{1}H-NMR (DMSO-d_6, 600 MHz) δ: 7.54 (dd, 2H, J = 6.5 Hz), 5.16-5.10 (m, 1H), 4.68 (dd, 1H, J = 9.1 Hz), 2.12 (d, 1H, J = 11.3 Hz), 1.96-1.82

(m, 3H), 1.78-1.62 (m, 3H), 1.61-1.38 (m, 8H), 1.35-1.21 (m, 4H), 1.17-1.08 (m, 1H), 1.07 (s, 3H), 1.05-0.96 (m, 1H), 0.92 (s, 10H), 0.84-0.80 (m, 6H), 0.76 (s, 3H). ^{13}C-NMR (DMSO-d$_6$, 151 MHz) δ: 178.3, 163.7, 162.5, 156.1, 153.7, 138.3, 122.1, 121.8, 120.3, 82.9, 54.5, 52.4, 46.9, 46.8, 41.7, 38.5, 38.4, 37.6, 37.4, 36.4, 36.3, 32.5, 30.2, 27.7, 27.6, 23.8, 23.3, 22.9, 22.7, 21.1, 17.8, 17.0, 16.9, 16.6, 15.1. IR (neat) v_{max}/cm^{-1} 2925 (m), 1718 (s), 1685 (s), 1473 (m), 1273 (s), 1252(s), 1139 (w), 1024 (w), 990 (s), 965 (s), 820 (m), 727 (m), 663 (w). HRMS-ESI m/z calcd for $C_{38}H_{48}O_6F_2$ (M+H)$^+$: 639.3503, found 639.3502.

(1S,2R,4aS,6aS,6bR,8aR,10S,12aR,12bR,14bR)-10-((2-Carboxy-4,5-difluorobenzoyl)oxy)-1,2,6a,6b,9,-9,12a-heptamethyl-1,3,4,5,6,6a,6b,7,8,8a,9,10,11,12,-12a,12b,13,-14b-octadecahydropicene-4a(2H)-carboxylic acid (37). Product **37** was obtained from ursolic acid (100.0 mg, 0.22 mmol, 1.00 eq) and 4,5-difluorophthalic anhydride (80.6 mg, 0.44 mmol, 2.00 eq) according to general procedure A. Reaction time: 9 days (100 °C). Purification: preparative HPLC (H_2O:MeCN 5% → 95% MeCN, solid phase material: C18). Yield: 40.8 mg (29%). HPLC (method B): t_R = 14.1 min, purity >95%. ^{1}H-NMR (DMSO-d$_6$, 600 MHz) δ: 7.70 (d, 1H, J = 7.4 Hz), 7.52 (d, 1H, J = 8.5 Hz), 5.25 (m, 1H), 4.73 (dd, 1H, J = 12.0, 4.7 Hz), 2.20 (d, 1H, J = 11.0 Hz), 2.06-1.97 (m, 1H), 1.97-1.82 (m, 4H), 1.75-1.63 (m, 5H), 1.59-1.47 (m, 4H), 1.39-1.28 (m, 4H), 1.17-1.07 (m, 5H), 1.06-1.00 (m, 1H), 0.96 (s, 6H), 0.93 (s, 3H), 0.90-0.86 (m, 4H), 0.85 (s, 3H), 0.79 (s, 3H). ^{13}C-NMR (DMSO-d$_6$, 151 MHz) δ: 178.3, 163.7, 162.5, 156.1, 153.7, 138.3, 124.5, 122.0, 121.8, 120.3, 82.9, 54.5, 52.4, 46.9, 46.8, 41.7, 38.5, 38.4, 37.6, 37.4, 32.5, 30.2, 27.7, 27.6, 23.8, 23.3, 22.9, 22.7, 21.1, 17.8, 17.0, 16.9, 16.6, 15.1. IR (neat) v_{max}/cm^{-1} 2924 (m), 1685 (s), 1606 (w), 1320 (m), 1253 (m), 1180 (m), 1116 (w), 965 (w), 899 (w), 734 (w). HRMS-ESI m/z calcd for $C_{38}H_{48}O_6F_2$ (M+H)$^+$: 639.3503, found 639.3502.

(1S,2R,4aS,6aS,6bR,8aR,10S,12aR,12bR,14bR)-10-((2-Carboxy-4,5-dimethoxybenzoyl)oxy)-1,2,6a,6b,-9,9,12a-heptamethyl-1,3,4,5,6,6a,6b,7,8,8a,9,10,11,-12,12a,12b,13,-14b-octadecahydropicene-4a(2H)-carboxylic acid (38). Product **38** was obtained from ursolic acid (200.0 mg, 0.44 mmol, 1.00 eq) and 5,6-dimethoxy-2-benzofuran-1,3-dione (182.3 mg, 0.88 mmol, 2.00 eq) according to general procedure A. Reaction time: 7 days (100 °C). Purification: preparative HPLC (H_2O:MeCN 5% → 95% MeCN, solid phase material: C18). Yield: 23.6 mg (8%). HPLC (method B): t_R = 12.2 min, purity >95%. ^{1}H-NMR ($CDCl_3$, 400 MHz) δ: 7.20 (s, 1H), 7.13 (s, 1H,), 5.17-5.13 (m, 1H), 4.62-4.56 (m, 1H), 3.84 (s, 3H), 3.83 (s, 3H), 2.18-2.06 (m, 1H), 2.01-1.75 (m, 5H), 1.75-1.67 (m, 2H), 1.76-1.59 (m, 2H), 1.59-1.39 (m, 8 H), 1.38-1.27 (m, 4H), 1.24 (s, 3H), 1.20-1.10 (m, 1H), 1.08 (s, 3H), 0.94 (s, 3H), 0.93 (s, 3H), 0.91 (s, 3H), 0.84 (s, 3H), 0.77 (s, 3H). ^{13}C-NMR ($CDCl_3$, 101 MHz) δ: 184.1, 170.6, 168.3, 151.3, 150.8, 138.0, 126.0, 125.9, 125.7, 124.0, 113.4, 112.1, 83.6, 56.2, 56.1, 55.3, 52.4, 48.0, 47.3, 41.9, 39.5, 39.0, 38.8, 38.1, 37.9, 36.9, 36.8, 32.7, 30.5, 29.6, 28.3, 28.0, 24.0, 23.6, 23.3, 22.8, 21.1, 18.1. IR (neat) v_{max}/cm^{-1} 2924 (m), 2850 (w), 1714 (w), 1694 (s), 1463 (w), 1353 (w), 1277 (s), 1205 (s), 1174 (s), 1136 (s), 986 (w), 949 (w), 703 (s), 647 (w). HRMS-ESI m/z calcd $C_{40}H_{56}O_8$ (M+Na)$^+$: 687.3867, found 687.3873.

(4aS,6aS,6bR,8aR,10S,12aR,12bR,14bS)-10-Hydroxy-2,2,6a,6b,9,9,12a-heptamethyl-N-(1H-tetrazol-5-yl)-1,3,4,5,6,6a,6b,7,8,8a,9,10,11,12,12a,12b,13,14b-octa-decahydropicene-4a(2H)-carboxamide (39). DIPEA (0.534 ml, 3.07 mmol, 14.00 eq) was added to a solution of oleanolic acid (100.0 mg, 0.22 mmol, 1.00 eq), 5-amino-1H-tetrazole monohydrate (99.3 mg, 0.96 mmol, 4.40 eq), HATU (99.9 mg, 0.26 mmol, 1.20 eq), and DMAP (2.70 mg, 0.02 mmol, 0.10 eq) in freshly degassed DMF (2.8 ml). After stirring for two days at room temperature, the solution was diluted with DCM (20.0 ml) and washed twice with H_2O (10.0 ml), sat. NaCl solution (10.0 ml), sat. $NaHCO_3$ solution (10.0 ml), sat. NaCl solution (10.0 ml) and H_2O (10.0 ml). The organic layer was dried over Na_2SO_4, filtered, and evaporated under reduced pressure. The crude product was purified by silica gel chromatography (Cyh:EtOAc 3:1, silica gel: 23 g) to afford pure compound **39** (25.9 mg, 23%). HPLC (method C): t_R = 5.7 min, purity >99%. ^{1}H-NMR ($CDCl_3$, 600 MHz) δ: 13.92 (s, 1H), 10.95 (s, 1H), 5.45 (t, 1H, J = 3.8 Hz), 3.21 (dd, 1H, J = 11.5, 4.4 Hz), 2.91 (t, 1H, J = 6.8 Hz), 2.29 (td, 1H, J = 14.3, 3.9 Hz), 2.06-2.00 (m, 1H), 1.99-1.84 (m, 3H), 1.78 (d, 1H, J = 13.6 Hz), 1.71 (td, 1H, J = 13.8, 4.1 Hz), 1.58-1.48 (m, 6H), 1.34-1.24 (m, 4H), 1.25-1.05 (m, 6H), 0.96 (s, 4H), 0.94 (s, 6H), 0.87 (s, 3H), 0.75 (s, 3H), 0.73-0.69 (m, 1H), 0.63 (s, 3H). ^{13}C-NMR (DMSO-d$_6$, 151 MHz) δ: 178.9, 150.7, 143.4, 123.5, 78.9, 55.1, 47.6, 47.5, 46.1, 41.8, 41.1, 39.2, 38.7, 38.4, 36.4, 33.9, 32.8, 32.4, 32.0, 31.4, 28.0, 27.3, 27.0, 25.8, 23.5, 23.4, 23.3, 18.2, 16.7, 15.5, 15.3. IR (neat) v_{max}/cm^{-1} 3194 (w), 2925 (s), 1668 (m), 1606 (m), 1582 (s), 1532 (w), 1463 (m), 1386 (m), 1028 (s), 996 (s), 920 (w), 655 (w). HRMS-ESI m/z calcd for $C_{31}H_{49}N_5O_2$ (M+Na)$^+$: 546.3778, found 546.3783.

*(4aS,6aS,6bR,8aR,10S,12aR,12bR,14bR)-N-(2-Amino-ethyl)-10-hydroxy-2,2,6a,6b,9,9,12a-heptamethyl-1,3,-4,5,6,6a,6b,7,8,8a,9,10,11,12,12a,12b,13,14b-octadeca-hydro-picene-4a(2H)-carboxamide (**40**).*[14] **Step 1:** DIPEA (1.34 ml, 7.66 mmol, 7.00 eq) was added to a solution of oleanolic acid (500.0 mg, 1.10 mmol, 1.00 eq), N-boc-ethylenediamine (0.38 ml, 2.40 mmol, 2.20 eq), HATU (499.5 mg, 1.31 mmol, 1.20 eq), and DMAP (13.4 mg, 0.11 mmol, 0.10 eq) in freshly degassed DMF (2.8 ml). The solution was stirred for 17 h at room temperature, diluted with DCM (70.0 ml), washed three times with H_2O (50.0 ml), sat. NaCl solution (50.0 ml), sat. $NaHCO_3$ solution (50.0 ml), sat. NaCl solution (50.0 ml), and H_2O (50.0 ml). After drying of the organic layer over Na_2SO_4, the solvent was removed under reduced pressure and the crude product was purified by silica gel chromatography (Cyh:EtOAc 3:1, silica gel: 32 g) to yield the pure intermediate 1 (592.7 mg, 90%). HPLC (method C): t_R = 8.4 min, purity >99%. ^{1}H-NMR ($CDCl_3$, 600 MHz) δ: 6.35 (t, 1H, J = 5.4 Hz), 5.41-5.33 (m, 1H), 3.61-3.41 (m, 1H), 3.27-3.17 (m, 3H), 3.16-3.10 (m, 1H), 2.54 (dd, 1H, J = 13.3, 4.4 Hz), 1.95-1.91 (m, 1H), 1.79-1.68 (m, 2H), 1.68-1.56 (m, 5H), 1.59-1.46 (m, 5H), 1.42 (s, 10H), 1.36-1.32 (m, 2H), 1.25-1.23 (m, 1H), 1.20-1.15 (m, 2H), 1.14 (s, 3H), 1.04-1.00 (m, 1H), 0.97 (s, 3H), 0.96-0.92 (m, 1H), 0.93-0.88 (m, 9H), 0.77 (s, 3H), 0.74 (s, 3H), 0.73-0.69 (m, 1H). ^{13}C-NMR ($CDCl_3$, 151 MHz) δ: 179.4, 156.9, 144.9, 123.3, 79.8, 79.2, 55.5, 48.0, 47.0, 46.6, 42.3, 40.8, 40.6, 39.7, 39.1, 38.8, 37.3, 34.5, 33.3, 33.0, 32.7, 31.0, 28.8, 28.4, 27.7, 27.5, 26.2, 24.0, 23.9, 18.6, 17.2, 15.9, 15.7. IR (neat) v_{max}/cm^{-1} 3551 (w), 2926 (s), 1692 (s), 1634 (m), 1519 (s), 1454 (m), 1388 (w), 1364 (s), 1251 (m), 1167 (s), 1029 (w), 997 (m). HRMS-ESI m/z calcd for $C_{37}H_{62}O_4N_2$ $(M+Na)^+$: 621.4602, found 621.4602. **Step 2:** The precursor from step 1(592.7 mg, 1.00 mmol, 1.00 eq) was dissolved in 4M HCl/dioxane (12.0 ml) and stirred for four hours at room temperature. The solvent was removed *in vacuo* and the crude product was purified by silica gel column chromatography (DCM:MeOH 1:1, silica gel: 32 g) to give product **40** (465.9 mg, 87%). HPLC (method D): t_R = 7.8 min, purity 95%. ^{1}H-NMR (DMSO-d_6, 400 MHz) δ: 8.12 (s, 1H), 7.68 (t, 1H, J = 5.6 Hz), 5.52-5.04 (m, 1H), 3.54-3.14 (m, 2H), 2.99 (dd, 1H, J = 9.6, 6.1 Hz), 2.90-2.65 (m, 3H), 1.91 (dt, 1H, J = 13.7, 6.7 Hz), 1.83-1.76 (m, 2H), 1.71-1.54 (m, 3H), 1.53-1.39 (m, 9H), 1.37-1.17 (m, 2H), 1.13-1.10 (m, 1H), 1.08 (s, 4H), 0.90-0.88 (m, 7H), 0.88-0.86 (m, 3H), 0.84 (s, 3H), 0.66 (s, 4H), 0.65 (s, 1H). ^{13}C-NMR (DMSO-d_6, 101 MHz) δ: 177.2, 143.9, 121.5, 76.8, 54.8, 47.1, 45.9, 45.3, 41.2, 38.5, 38.4, 38.1, 36.7, 36.6, 36.6, 33.6, 33.0, 32.5, 32.3, 30.4, 28.2, 27.0, 25.7, 23.4, 22.9, 22.2, 18.0, 16.8, 16.0, 15.1. IR (neat) v_{max}/cm^{-1} 3394 (w), 2928 (s), 1737 (w), 1639 (s), 1547 (m), 1464 (m), 1391 (w), 1240 (w), 1169 (w), 1080 (w), 1044 (s), 997 (m). HRMS-ESI m/z calcd for $C_{32}H_{55}O_2N_2$ $(M+Na)^+$: 499.4258, found 499.4259.

*4,5-Difluoro-2-((((3S,4aR,6aR,6bS,8aS,12aS,14aR,-14bR)-8a-(hydroxycarbamoyl)-4,4,6a,6b,11,11,14b-heptamethyl-1,2,3,4,4a,5,6,6a,6b,7,8,8a,9,10,11,12,-12a,14,14a,14b-icosahydropicen-3-yl)oxy)carbonyl)-benzoic acid (**41**).* **Step 1:** NEt_3 (0.317 ml, 1.86 mmol, 1.70 eq) and DMAP (13.4 mg, 0.11 mmol, 0.10 eq) were added at room temperature to a solution of oleanolic acid (500.0 mg, 1.09 mmol, 1.00 eq) and acetic anhydride (0.232 ml, 2.19 mmol, 2.00 eq) in dry pyridine (6.6 ml). The reaction mixture was stirred for 17 h. Then, the solution was diluted with DCM (20.0 ml) and washed three times with saturated NaCl solution (10.0 ml), saturated $NaHCO_3$ solution (10.0 ml), and saturated NaCl solution (10.0 ml). After drying of the organic layer over Na_2SO_4 and removal of the solvent under reduced pressure, the crude product was purified by reversed phase column chromatography (MeOH:H_2O 70% → 95% MeCN, solid phase material: C18, 51 g) to afford the precursor 1 (349.5 mg, 64%). HPLC (method B): t_R = 13.7 min, purity >95%. ^{1}H-NMR ($CDCl_3$, 400 MHz) δ: 5.28-5.25 (m, 1H), 4.52-4.46 (m, 1H), 2.81 (dd, 1H, J = 14.0, 4.6 Hz), 2.04 (s, 3H), 2.02-1.92 (m, 1H), 1.91-1.84 (m, 2H), 1.81-1.65 (m, 2H), 1.65-1.50 (m, 8H), 1.47-1.19 (m, 6H), 1.12 (s, 3H), 1.10-1.00 (m, 2H), 0.93 (s, 3H), 0.92 (s, 3H), 0.90 (s, 3H), 0.86 (s, 3H), 0.84 (s, 3H), 0.84-0.81 (m, 1H), 0.74 (s, 3H). ^{13}C-NMR ($CDCl_3$, 101 MHz) δ: 184.2, 171.0, 143.6, 122.5, 80.9, 55.3, 47.5, 46.5, 45.8, 41.5, 40.9, 39.2, 38.0, 37.7, 37.0, 33.8, 33.0, 32.5, 30.6, 28.0, 27.6, 25.9, 23.6, 23.5, 23.4, 22.8, 21.3. IR (neat) v_{max}/cm^{-1} 2926 (s), 1725 (m), 1686 (m), 1462 (w), 1371 (m), 1242 (s), 1159 (w), 1026 (m), 736 (w). HRMS-ESI m/z calcd for $C_{32}H_{50}O_4$ $(M+Na)^+$: 521.3601, found 521.3600. **Step 2:** Oxalylchloride (0.361 ml, 4.20 mmol, 6.00 eq) was added at 0 °C to a solution of the precursor of step 1 (349.5 mg, 0.70 mmol, 1.00 eq) in DCM (18.0 ml). The solution was allowed to warm up to room temperature and stirred for two hours. Then, the solvent was removed *in vacuo* and the remaining residue was dissolved in DCM (22.0 ml). NEt_3 (0.684 ml, 4.91 mmol, 7.00 eq) and hydroxylamine hydrochloride (379.8 mg, 5.47 mmol, 7.80 eq) were added and the reaction mixture was stirred for 3 h at room temperature. The solution was washed three times with saturated NaCl solution (10.0 ml), saturated $NaHCO_3$ solution (10.0 ml), H_2O (10.0 ml), and saturated NaCl solution (10.0 ml). Additionally, the aqueous layer was extracted once with DCM (20.0 ml). The combined organic layers were dried over Na_2SO_4, followed by solvent evaporation under reduced pressure. The crude product was purified by silica gel column chromatography (Cyh:EtOAc 4:1, silica gel: 37 g) to obtain pure intermediate 2 (131.8 mg, 37%). HPLC

(method B): t_R = 12.4 min, purity >99%. ^{1}H-NMR ($CDCl_3$, 600 MHz) δ: 5.48-5.34 (m, 1H), 4.53-4.42 (dd, 1H, J = 9.6, 6.2 Hz), 2.50-2.41 (m, 1H), 2.04 (s, 3H), 2.02-1.88 (m, 3H), 1.82-1.71 (m, 2H), 1.64-1.55 (m, 6H), 1.49-1.30 (m, 4H), 1.27-1.17 (m, 3H), 1.16 (s, 3H), 1.09-0.99 (m, 2H), 0.94 (s, 3H), 0.90 (s, 3H), 0.88 (s, 3H), 0.86 (s, 6H), 0.85-0.83 (m, 1H), 0.81 (s, 3H). ^{13}C-NMR ($CDCl_3$, 151 MHz) δ: 171.0, 144.8, 123.8, 80.8, 55.2, 47.4, 46.2, 41.9, 40.7, 39.4, 38.2, 37.7, 36.8, 33.9, 32.9, 32.1, 31.9, 30.7, 28.0, 27.2, 25.7, 23.7, 23.6, 23.5, 23.4, 21.3. IR (neat) v_{max}/cm^{-1} 3399 (w), 2940 (s), 1719 (m), 1638 (s), 1463 (m), 1370 (m), 1242 (s), 1159 (w), 1026 (m), 729 (w). HRMS-ESI m/z calcd for $C_{32}H_{51}NO_4$ $(M+Na)^+$: 536.3710, found 536.3746. **Step 3:** Intermediate 2 (102.5 mg, 0.20 mmol, 1.00 eq) was added to a solution of KOH (106.3 mg, 1.90 mmol, 9.50 eq) in MeOH (11.0 ml). The solution was stirred for two days at room temperature. Then, the solvent was removed *in vacuo*. The remaining residue was dissolved in EtOAc (20.0 ml) and washed three times with H_2O (10.0 ml). The organic phase was dried over Na_2SO_4 followed by evaporation of the solvent under reduced pressure. The crude product was purified by reversed phase column chromatography (MeOH:H_2O 70% → 95%, solid phase material: C18, 32 g) to afford pure intermediate 3 (89.1 mg, 95%). HPLC (method B): t_R = 10.6 min, purity >99%. ^{1}H-NMR ($CDCl_3$, 400 MHz) δ: 5.48-5.38 (m, 1H), 3.26-3.18 (m, 1H), 2.03-1.95 (m, 3H), 1.89-1.84 (m, 1H), 1.80-1.74 (m, 1H), 1.68-1.37 (m, 11H), 1.33-1.27 (m, 3H), 1.25 (s, 3H), 1.11 (s, 3H), 1.08-1.04 (m, 1H), 1.03-1.00 (m, 1H), 0.99 (s, 3H), 0.95 (s, 3H), 0.92 (s, 3H), 0.91-0.88 (m, 1H), 0.86 (d, 3H, J = 6.5 Hz), 0.79 (s, 6H). ^{13}C-NMR ($CDCl_3$, 101 MHz) δ: 177.2, 140.4, 126.7, 79.0, 55.1, 52.0, 47.5, 46.9, 42.4, 39.5, 39.4, 39.0, 38.7, 38.6, 36.9, 36.6, 32.4, 31.9, 30.6, 29.7, 28.1, 27.7, 24.8 23.6, 23.3, 22.7, 21.3. IR (neat) v_{max}/cm^{-1} 3379 (w), 2925 (s), 2871 (w), 1725 (s), 1686 (w), 1462 (m), 1385 (w), 1275 (s), 1123 (m), 1028 (w), 749 (s). HRMS-ESI m/z calcd for $C_{30}H_{48}O_3$ $(M+Na)^+$: 470.3640, found 470.3642. **Step 4:** A reaction mixture consisting of the product of the preceding reaction (69.7 mg, 0.15 mmol, 1.00 eq), 3,6-difluorophthalic anhydride (54.4 mg, 0.30 mmol, 2.00 eq), and DMAP (1.8 mg, 0.01 mmol, 0.10 eq) in pyridine (2.5 ml) was stirred for three days at 110 °C. After that time, the solution was diluted with EtOAc (10.0 ml) and washed three times with 1 M HCl (15.0 ml) and H_2O (15.0 ml). The aqueous layer was extracted three times with EtOAc (20.0 ml). The combined organic layers were dried over Na_2SO_4, filtered and evaporated under reduced pressure. The crude product was purified by preparative HPLC (MeCN:H_2O 5% → 95% MeCN, solid phase material: C18) to obtain product **41** (27.2 mg, 29%). HPLC (method B): t_R = 12.2 min, purity >95%. ^{1}H-NMR ($CDCl_3$, 600 MHz) δ: 7.70-7.59 (m, 2H), 5.52-5.45 (m, 1H), 4.54 (dd, 1H, J = 11.81, 4.66 Hz), 2.46 (dd, 1H, J = 13.01, 4.31 Hz), 2.01-1.96 (m, 1H), 1.95-1.88 (m, 1H), 1.84-1.71 (m, 3H), 1.70-1.53 (m, 6H), 1.52-1.30 (m, 4H), 1.30-1.21 (m, 3H), 1.17 (s, 3H), 1.15-1.00 (m, 3H), 0.98 (s, 3H), 0.93 (s, 3H), 0.91 (s, 3H), 0.90-0.84 (m, 7H), 0.80 (s, 3H). ^{13}C-NMR ($CDCl_3$, 151 MHz) δ: 176.9, 169.9, 165.4, 150.7, 144.7, 129.7, 124.2, 119.8, 119.2, 84.1, 55.1, 46.9, 46.0, 45.4, 41.6, 41.1, 39.3, 37.7, 37.7, 36.9, 33.7, 32.8, 32.0, 31.6, 30.60, 28.4, 27.30, 25.6, 23.4, 21.9, 18.2, 17.3, 16.6, 16.0. IR (neat) v_{max}/cm^{-1} 3358 (w), 2945 (m), 1723 (m), 1698 (m), 1603 (m), 1442 (m), 1323 (s), 1280 (s), 1182 (s), 1113 (m), 965 (w), 796 (m), 653 (w). HRMS-ESI m/z calcd for $C_{38}H_{50}F_2NO_6$ $(M-H)^-$: 654.3612, found 654.3611.

*(4aS,6aR,6bR,8aR,10S,12aS,12bR,14bS)-10-((4,5-Difluoro-2-(hydroxycarbamoyl)benzoyl)oxy)-2,2,6a,-6b,9,9,12a,12b-octamethyl-1,3,4,5,6,6a,6b,7,8,8a,9,10,-11,12,12a,12b,13,14b-octadecahydropicene-4a(2H)-carboxylic acid (**42**).* Oxalylchloride (1.260 ml, 14.69 mmol, 12.00 eq) was added to a solution of compound **43** (784.0 mg, 1.22 mmol, 1.00 eq) in DCM (20.0 ml) at 0 °C. The solution was allowed to warm up to room temperature and stirred for two hours. Then, the solvent was removed *in vacuo* and the remaining residue was dissolved in DCM (35.0 ml). NEt_3 (2.390 ml, 17.14 mmol, 14.00 eq) and hydroxylamine hydrochloride (1.36 g, 19.58 mmol, 16.00 eq) were added and the reaction was stirred for three hours at room temperature. The solution was washed three times with saturated NaCl solution (20.0 ml), saturated $NaHCO_3$ solution (20.0 ml), H_2O (20.0 ml), and saturated NaCl solution (20.0 ml). The aqueous layer was extracted once with DCM (20.0 ml). The combined organic layers were dried over Na_2SO_4, followed by solvent evaporation under reduced pressure. The crude product was purified by preparative HPLC (MeCN:H_2O 5% → 95% MeCN, solid phase material: C18) to afford product **42** (17.5 mg, 2%). HPLC (method B): t_R = 11.7 min, purity >95%. ^{1}H-NMR ($CDCl_3$, 400 MHz) δ: 7.87 (dd, 1H, J = 10.5, 7.6 Hz), 7.36 (dd, 1H, J = 9.4, 7.1 Hz), 5.36-5.20 (m, 1H), 4.74 (dd, 1H, J = 11.8, 4.5 Hz), 2.96-2.80 (m, 1H), 2.12-1.78 (m, 4H), 1.76-1.45 (m, 7H), 1.44-1.29 (m, 2H), 1.28-1.16 (m, 3H), 1.13 (s, 4H), 1.08-1.05 (m, 1H), 1.02 (s, 3H), 1.01-0.92 (m, 7H), 0.91 (s, 3H), 0.87 (s, 4H), 0.70 (s, 3H). ^{13}C-NMR ($CDCl_3$, 101 MHz) δ: 185.2, 166.4, 164.0, 143.6, 122.8, 85.8, 55.3, 47.2, 46.5, 45.6, 41.2, 41.0, 39.2, 37.9, 37.7, 37.2, 33.6, 33.1, 32.8, 32.0, 30.7, 28.5, 27.6, 26.2, 23.6, 22.3, 23.2, 22.9, 18.2, 17.2, 15.6. IR (neat) v_{max}/cm^{-1} 2949 (w), 1689 (w), 1463 (w), 1275 (m), 1267 (m), 1261 (m), 1185 (w), 1133 (w), 1066 (w), 964 (w), 897 (w), 764 (s), 745 (s). HRMS-ESI m/z calcd for $C_{38}H_{51}F_2NO_6$ $(M+Na)^+$: 678.3577, found 678.3579.

*(4aS,6aS,6bR,8aR,10S,12aR,12bR,14bR)-10-((2-Carboxy-4,5-difluorobenzoyl)oxy)-2,2,6a,6b,9,9,12a-heptamethyl-1,3,4,5,6,6a,6b,7,8,8a,9,10,11,12,12a,12-b,13,14b-octa-decahydropicene-4a(2H)-carboxylic acid (**43**).* Product **43** was obtained from oleanolic acid (500.0 mg, 1.09 mmol, 1.00 eq) and 4,5-difluorophthalic anhydride (806.2 mg, 4.38 mmol, 4.00 eq) according to general procedure A. Reaction time: 20 h (110 °C). Purification: silica gel chromatography (Cyh:EtOAc 4:1, silica gel: 32 g). Yield: 330.6 mg (47%). HPLC (method C): t_R = 8.8 min, >95%. ^{1}H-NMR (DMSO-d_6, 400 MHz) δ: 7.85-7.76 (m, 1H), 7.77-7.68 (m, 1H), 5.20-5.14 (m, 1H), 4.66-4.57 (m, 1H), 2.75 (dd, 1H, J = 13.7, 4.6 Hz), 2.02-1.79 (m, 3H), 1.74-1.56 (m, 6H), 1.56-1.39 (m, 5H), 1.37-1.14 (m, 5H), 1.12 (s, 3H), 1.10-0.96 (m, 3H), 0.91 (s, 6H), 0.88 (s, 6H), 0.82 (s, 3H), 073 (s, 3H). ^{13}C-NMR (DMSO-d_6, 101 MHz) δ: 178.6, 166.3, 165.3, 143.9, 121.4, 118.6, 82.4, 54.7, 46.8, 45.7, 45.8, 41.4, 40.8, 37.5, 36.5, 33.3, 32.8, 32.2, 32.1, 30.4, 27.9, 27.2, 25.6, 23.4, 22.9, 22.6, 22.5, 17.8, 16.8, 16.7, 15.0. IR (neat) v_{max}/cm^{-1} 2945 (m), 1839 (w), 1710 (s), 1604 (w), 1521 (w), 1439 (m), 1389 (w), 1322 (s), 1304 (m), 1184 (s), 1117 (m), 1116 (m), 963 (m), 767 (m). HRMS-ESI m/z calcd for $C_{38}H_{50}O_6F_2$ $(M+Na)^+$: 663.3468, found 663.3468.

*(4aS,6aS,6bR,8aR,10S,12aR,12bR,14bR)-10-((2-Carboxy-3,6-difluorobenzoyl)oxy)-2,2,6a,6b,9,9,12a-heptamethyl-1,3,4,5,6,6a,6b,7,8,8a,9,10,11,12,12a,-12b,13,14b-octa-decahydropicene-4a(2H)-carboxylic acid (**44**).* Product **44** was obtained from oleanolic acid (100.0 mg, 0.22 mmol, 1.00 eq) and 3,6-difluorophthalic anhydride (80.6 mg, 0.44 mmol, 2.00 eq) according to general procedure A. Reaction time: 3 days (100 °C). Purification: preparative TLC (DCM:MeOH 96:4). Yield: 34.3 mg (24%). HPLC (method C): t_R = 8.1 min, purity >95%. ^{1}H-NMR (DMSO-d_6, 400 MHz) δ: 7.54 (dd, 2H, J = 6.5 Hz,) 5.69-4.89 (m, 1H), 4.68 (dd, 1H, J = 9.2, 7.2 Hz), 2.75 (dd, 1H, J = 13.9, 4.7 Hz), 1.92 (dt, 1H, J = 13.5, 4.0 Hz), 1.85-1.81 (m, 2H), 1.71-1.56 (m, 7H), 1.53-1.42 (m, 4H), 1.37-1.29 (m, 2H), 1.28-1.22 (m, 1H), 1.16-1.14 (m, 1H), 1.13 (s, 3H), 1.10-1.04 (m, 2H), 1.03-0.98 (m, 1H), 0.92 (s, 3H), 0.88 (s, 6H), 0.81 (s, 3H), 074 (s, 3H). ^{13}C-NMR (DMSO-d_6, 101 MHz) δ: 178.5, 163.7, 162.5, 155.7, 154.0, 143.9, 121.8, 121.4, 120.2, 82.8, 54.5, 46.8, 45.7, 45.5, 41.4, 40.8, 40.0, 38.9, 37.4, 37.3, 36.5, 33.3, 32.8, 32.2, 32.1, 27.6, 27.2, 25.6, 23.4, 22.9, 22.6, 17.8, 16.8, 16.6, 15.0. IR (neat) v_{max}/cm^{-1} 2936 (m), 2868 (w), 1724 (s), 1455 (m), 1369 (w), 1236 (s),1125 (w), 975 (w), 818 (w), 729 (m). HRMS-ESI m/z calcd for $C_{38}H_{50}O_6F_2$ $(M+Na)^+$: 663.3468, found 663.3468.

*2-((((3S,4aR,6aR,6bS,8aS,12aR,14aR,14bR)-8a-Car-boxy-4,4,6a,6b,11,11,14b-heptamethyl-1,2,3,4,4a,5,6,-6a,6b,7,8,-8a,9,10,11,12,12a,14,14a,14b-icosahydro-picen-3-yl)oxy)-carbonyl)thiophene-3-car-boxylic acid (**45**).* Compound **45** was isolated as a regioisomer of compound **46**. Yield: 24.7 mg (18%). HPLC (method C): t_R = 9.6 min, purity >95%. ^{1}H-NMR (DMSO-d_6, 400 MHz) δ: 7.82 (d, 1H, J = 5.1 Hz), 7.27 (d, 1H, J = 5.1 Hz), 5.17 (t, 1H, J = 3.7 Hz), 4.63 (dd, 1H, J = 10.0, 6.4 Hz), 2.80-2.69 (m, 1H), 1.99-1.85 (m, 1H), 1.85-1.78 (m, 1H), 1.73-1.53 (m, 6H), 1.52-1.41 (m, 4H), 1.41-1.29 (m, 3H), 1.25-1.22 (m, 2H), 1.19-1.14 (m, 1H), 1.12 (s, 3H), 1.08-0.97 (m, 3H), 0.92 (m, 3H), 0.90 (s, 3H), 0.87 (s, 7H), 0.81 (s, 3H), 073 (s, 3H). ^{13}C-NMR (DMSO-d_6, 101 MHz) δ: 178.5, 163.9, 162.1, 143.8, 136.8, 131.1, 128.2, 121.4, 81.7, 54.6, 46.8, 45.7, 45.4, 41.4, 40.8, 40.0, 38.9, 37.4, 36.5, 33.3, 32.8, 32.2, 32.1, 30.4, 29.0, 27.8, 27.2, 25.6, 23.4, 22.0, 17.8, 16.8, 16.6, 15.0. IR (neat) v_{max}/cm^{-1} 2925 (m), 2851 (w), 2663 (w), 1687 (m), 1649 (m), 1524 (w),1442 (m), 1293 (s), 1270 (w), 987 (m), 729 (m). HRMS-ESI m/z calcd for $C_{36}H_{50}O_6S$ $(M+Na)^+$: 633.3220, found 633.3221.

*3-((((3S,4aR,6aR,6bS,8aS,12aR,14aR,14bR)-8a-Car-boxy-4,4,6a,6b,11,11,14b-heptamethyl-1,2,3,4,4a,5,-6,6a,6b,7,8,8a,9,10,11,12,12a,14,14a,14b-icosahydro-picen-3-yl)oxy)-carbonyl)thiophene-2-carboxylic acid (**46**).* Product **46** was obtained from oleanolic acid (100.0 mg, 0.22 mmol, 1.00 eq) and 2,3-thiophene dicarboxylic anhydride (135.0 mg, 0.88 mmol, 4.00 eq) according to general procedure A. Reaction time: 5 days (100 °C). Purification: preparative TLC (DCM:MeOH 96:4). Yield: 15.3 mg (11%). HPLC (method C): t_R = 9.3 min, purity >95%. ^{1}H-NMR (DMSO-d_6, 400 MHz) δ: 7.82 (d, 1H, J = 5.0 Hz), 7.24 (d, 1H, J = 5.1 Hz), 5.17 (t, 1H, J = 7.6 Hz), 4.56 (dd, 1H, J = 11.4, 4.9 Hz), 2.75 (dd, 1H, J = 14.0, 4.7 Hz), 1.95-1.88 (m, 1H), 1.87-1.80 (m, 1H), 1.73-1.54 (m, 6H), 1.53-1.42 (m, 4H), 1.41-1.28 (m, 3H), 1.27-1.21 (m, 2H), 1.20-1.13 (m, 1H), 1.12 (s, 3H), 1.09-0.96 (m, 3H), 0.95-0.91(m, 4H), 0.89 (s, 3H), 0.88 (s, 9H), 0.73 (s, 3H). ^{13}C-NMR (DMSO-d_6, 101 MHz) δ: 178.6, 160.8, 143.9, 131.8, 128.6, 121.4, 121.4, 82.1, 54.5, 46.8, 45.7, 45.5, 41.4, 40.8, 40.0, 38.9, 37.6, 36.5, 33.3, 32.8, 32.2, 32.1, 30.4, 29.0, 27.8, 25.6, 23.4, 23.0, 17.8, 16.8, 16.6, 15.0. IR (neat) v_{max}/cm^{-1} 2948 (m), 2668 (w), 1738 (m), 1688 (m), 1633 (w), 1423 (m), 1270 (s), 1265 (w), 1114 (w), 907 (w), 726 (s), 651 (w). HRMS-ESI m/z calcd for $C_{36}H_{50}O_6S$ $(M+Na)^+$: 633.3220, found 633.3223.

(4aS,6aS,6bR,8aR,10S,12aR,12bR,14bR)-10-((2-Carboxy-4,5-dimethoxybenzoyl)oxy)-2,2,6a,6b,9,9,-12a-heptamethyl-1,3,4,5,6,6a,6b,7,8,8a,9,10,11,12,-12a,12b,13,14b-octa-decahydropicene-4a(2H)-car-boxylic acid (47). Product **47** was obtained from oleanolic acid (200.0 mg, 0.44 mmol, 1.00 eq) and 5,6-dimethoxy-2-benzofuran-1,3-dione (136.7 mg, 0.66 mmol, 1.50 eq) according to general procedure A. Reaction time: 7 days (100 °C). Purification: preparative HPLC (H_2O:MeCN 5% → 95% MeCN, solid phase material: C18). Yield: 32.2 mg (11%). HPLC (method B): t_R = 12.1 min, purity >95%. ^{1}H-NMR (acetone-d_6/acetic acid-d_4, 400 MHz) δ: 7.21 (s, 1H), 7.12 (s, 1H), 5.19-5.16 (m, 1H), 4.63-4.55 (m, 1H), 2.80-2.71 (m, 1H), 1.97-1.88 (m, 1H), 1.88-1.80 (m, 2H), 1.74-1.64 (m, 3H), 1.64-1.55 (m, 4H), 1.54-1.40 (m, 4H), 1.38-1.20 (m, 4H), 1.18-1.14 (m, 1H), 1.13 (s, 3H), 1.10-0.96 (m, 3H), 0.94 (s, 3H), 0.90 (s, 3H), 0.88 (s, 6H), 0.83 (s, 3H), 0.74 (s, 3H). ^{13}C-NMR (acetone-d_6/acetic acid-d_4, 101 MHz) δ: 181.4, 169.8, 168.1, 152.0, 151.6, 144.7, 127.2, 125.6, 123.1, 112.8, 112.5, 83.2, 56.4, 56.4, 56.2, 48.3, 47.1, 46.7, 42.5, 42.1, 40.2, 38.8, 38.5, 37.7, 34.4, 33.4, 33.3, 31.2, 30.3, 28.5, 28.4, 26.2, 24.1, 23.8, 23.7, 23.6, 18.9, 17.5, 17.2, 15.7. IR (neat) v_{max}/cm^{-1} 2941 (m), 2626 (w), 1736 (m), 1691 (s), 1598 (w), 1373 (w), 1292 (s), 1213 (s), 1179 (m), 1137 (m), 1059 (s), 986 (w), 876 (w), 767 (w), 633 (w). HRMS-ESI m/z calcd for $C_{40}H_{56}O_8$ $(M+Na)^+$: 687.3867, found 687.3862.

(4aS,6aS,6bR,8aR,10S,12aR,12bR,14bS)-10-((2-(methoxy-carbonyl)benzoyl)oxy)-2,2,6a,6b,9,9,12a-hepta-methyl-1,3,4,5,6,6a,6b,7,8,8a,9,10,11,12,12a,-12b,13,14b-octa-decahydropicene-4a(2H)-carboxylic acid (48). **Step 1:** 2-(methoxycarbonyl)benzoic acid (500.0 mg, 2.78 mmol, 1.00 eq) was dissolved in DCM (9.0 ml) and cooled down to 0 °C. Oxalylchloride (0.476 ml, 5.55 mmol, 2.00 eq) and two drops DMF were added and the solution was stirred for 15 h under argon atmosphere. The solvent was removed under reduced pressure and the crude product was used without further purification in the second step**. Step 2:** The crude product of the first step (547.9 mg, 2.76 mmol, 4.20 eq) was dissolved in DCM (5.0 ml) and a solution of oleanolic acid (300.0 mg, 2.76 mmol, 1.00 eq) and DMAP (8.0 mg, 0.07 mmol, 0.10 eq) in pyridine (5.0 ml) were slowly added by a dropping funnel. After stirring for 17 h at room temperature, the solvent was removed under reduced pressure and H_2O (20.0 ml) was added to the residue. The suspension was stirred for additional 20.0 min at room temperature. The precipitate was filtered off and the resulting crude product was purified by reversed phase column chromatography (MeOH:H_2O 50% → 95%, solid phase material: C18, 32 g) to afford pure compound **48** (420.5 mg, 68%). HPLC (method B): t_R = 14.7 min, purity >95%. ^{1}H-NMR (CDCl3, 400 MHz) δ: 7.82-7.78 (m, 1H), 7.75-7.66 (m, 3H), 7.62-7.57 (m, 2H), 7.55-7.50 (m, 2H), 5.33 (t, 1H, J = 3.7 Hz), 4.75 (dd, 1H, J = 11.8, 4.6 Hz), 3.91 (s, 3H), 3.89 (s, 3H), 2.85 (dd, 1H, J = 13.6, 4.6 Hz), 2.15-1.99 (m, 1H), 1.90-1.82 (m, 3H), 1.77-1.55 (m, 10H), 1.48-1.25 (m, 6H), 1.16 (s, 3H), 1.14-1.04 (m, 2H), 0.96 (s, 6H), 0.93 (s, 3H), 0.92 (s, 3H), 0.91 (s, 3H), 0.89-0.86 (m, 1H), 0.79 (s, 3H). ^{13}C-NMR ($CDCl_3$, 101 MHz) δ: 172.3, 168.3, 167.3, 167.1, 163.4, 142.8, 132.5, 132.2, 131.8, 131.4, 130.9,130.9, 129.4, 129.1, 128.8, 128.8, 123.1, 82.5, 55.5, 52.9, 52.6, 48.4, 47.6, 45.8, 41.8, 41.2, 39.4, 38.2, 38.0, 36.9, 33.7, 32.9, 32.7, 31.3, 30.6, 28.1, 27.7, 25.7, 23.5, 23.5, 23.3, 23.1, 18.2, 17.0, 16.8, 15.5. IR (neat) v_{max}/cm^{-1} 2947 (m), 1728 (s), 1695 (s), 1599 (w), 1580 (w), 1433 (w), 1278 (s), 1123 (m), 1072 (m), 994 (w), 742 (m), 706 (w). HRMS-ESI m/z calcd for $C_{39}H_{54}O_6$ $(M-H)^-$: 617.3848, found 617.3848.

(4aR,6aR,6bS,8aS,12aS,12bS,13S,14aR,14bR)-13-Hydroxy-4,4,6a,6b,11,11,14b-heptamethyloctadeca-hydro-3H,9H-12b,8a-(epoxymethano)picene-3,16-dione (49).[15] A solution of oleanolic acid (2.0 g, 4.38 mmol, 1.00 eq) in DCM (14.3 ml) and MeOH (2.8 ml) was cooled down to -50 °C. Ozone gas was passed through the solution for three hours. After completion of the reaction, excess ozone was removed by passing nitrogen gas through the reaction vessel until the blue color had disappeared. Solvent evaporation under reduced pressure and recrystallization from iPrOH afforded the product **49** (1.594 g, 77%). HPLC (method C): t_R = 6.4 min, purity 94% (poor solubility in MeCN/H_2O). ^{1}H-NMR ($CDCl_3$, 400 MHz) δ: 3.90 (t, 1H, J = 2.9 Hz), 2.48-2.37 (m, 1H), 2.57-2.48 (m, 1H), 2.20-2.09 (m, 2H), 2.08-1.99 (m, 3H), 1.99-1.84 (m, 2H), 1.72 (dd, 1H, J = 13.2, 2.3 Hz), 1.66-1.51 (m, 4H), 1.50-1.43 (m, 3H), 1.42-1.34 (m, 3H), 1.31 (s, 3H), 1.30-1.24 (m, 3H), 1.19 (s, 3H), 1.09 (s, 3H), 1.04 (s, 3H), 0.98 (s, 3H), 0.90 (s, 3H). ^{13}C-NMR ($CDCl_3$, 101 MHz) δ: 217.5, 179.7, 90.5, 76.2, 54.8, 51.1, 47.3, 44.7, 43.8, 42.1, 39.5, 39.4, 36.1, 34.1, 33.9, 33.3, 33.2, 31.5, 29.1, 28.0, 27.4, 26.6, 23.8, 21.1, 21.0, 19.0, 18.3, 18.1, 16.2. IR (neat) v_{max}/cm^{-1} 3508 (m), 2948 (s), 2919 (s), 2891 (w), 2868 (w), 1734 (s), 1705 (s), 1626 (m), 1453 (w), 1387 (m), 1256 (m), 1226 (w), 1142 (w), 1068 (m), 909 (w), 717 (w). HRMS-ESI m/z calcd for $C_{30}H_{48}O_4$ $(M+Na)^+$: 495.3445, found 495.3444.

(3S,4aR,6aR,6bS,8aS,12aS,12bS,13S,14aR,14bR)-3,-13-Dihydroxy-4,4,6a,6b,11,11,14b-heptamethylocta-decahydro-1H,9H-12b,8a-(epoxymethano)picen-16-one ***(50)***. $NaBH_4$ (2.143 g, 56.63 mmol, 17.00 eq) was added to a solution of 49 (1.568 g, 3.33 mmol, 1.00 eq) in a 1:1 mixture of DCM/MeOH (260.0 ml) at -78 °C. After one hour at -78 °C the solution was allowed to warm up to room temperature and stirred for additional three days. The reaction was quenched with 1 M NaOH solution (17.0 ml) and the aqueous layer was extracted three times with DCM (20.0 ml). The organic layer was dried over Na_2SO_4, followed by evaporation of the solvent under reduced pressure to give pure product **50** (1.444 g, 92%). HPLC (method C): t_R = 5.6 min, purity >95% (poor solubility in MeCN/H_2O). ^{1}H-NMR ($CDCl_3$, 600 MHz) δ: 3.88 (t, 1H, J = 2.9 Hz), 3.22 (dd, 1H, J = 11.6, 4.7 Hz), 2.13 (td, 1H, J = 13.3, 5.8 Hz), 2.07-1.94 (m, 3H), 1.92-1.82 (m, 2H), 1.72 (dt, 1H, J = 13.0, 3.7 Hz), 1.67-1.33 (m, 11H), 1.30 (s, 3H), 1.28-1.21 (m, 2H), 1.17 (m, 1H), 1.14 (s, 3H), 0.99 (s, 3H), 0.98 (s, 3H), 0.95 (m, 1H), 0.90 (s, 3H), 0.88 (s, 3H), 0.78 (s, 3H), 0.76-0.73 (m, 1H). ^{13}C-NMR ($CDCl_3$, 151 MHz) δ: 180.0, 90.6, 78.8, 76.4, 55.2, 51.1, 44.7, 44.6, 42.3, 42.1, 39.4, 38.8, 36.4, 34.1, 34.0, 33.3, 31.6, 28.8, 28.0, 28.0, 27.5, 27.2, 23.9, 21.2, 18.6, 18.5, 17.7, 16.3, 15.3. IR (neat) v_{max}/cm^{-1} 3555 (w), 2945 (m), 2865 (w), 2599 (w), 1733 (s), 1695 (m), 1469 (w), 1258 (w),1061 (m), 946 (m), 907 (w), 898 (w). HRMS-ESI m/z calcd for $C_{30}H_{48}O_4$ $(M+Na)^+$: 495.3445, found 495.3444.

*4,5-Difluoro-2-(((3S,4aR,6aR,6bS,8aS,12aS,12bS,-13S,14aR,14bR)-13-hydroxy-4,4,6a,6b,11,11,14b-heptamethyl-16-oxooctadecahydro-1H,9H-12b,8a-(epoxymethano)picen-3-yl)oxy)carbonyl)benzoic acid (**51**)*. Product **51** was obtained from compound **50** (100.0 mg, 0.22 mmol, 1.00 eq) and 4,5-difluorophthalic anhydride (80.6 mg, 0.44 mmol, 2.00 eq) according to general procedure A. Reaction time: 3 days (110 °C). Purification: silica gel chromatography (DCM:MeOH 95:5, silica gel: 23 g). Yield: 40.2 mg (28%). HPLC (method C): t_R = 8.3 min, >95%. ^{1}H-NMR (DMSO-d_6, 600 MHz) δ: 7.58 (m, 1H), 7.34 (m, 1H), 4.56 (dd, 1H, J = 11.9, 4.5 Hz), 3.67 (m, 1H), 2.15 (td, 1H, J = 13.3, 6.0 Hz), 2.00-1.87 (m, 3H), 1.85-1.77 (m, 1H), 1.77-1.52 (m, 6H), 1.53-1.43 (m, 3H), 1.38 (m, 3H), 1.27 (s, 3H), 1.25-1.20 (m, 1H), 1.20-1.14 (m, 1H), 1.13-1.07 (m, 2H), 1.03 (s, 3H), 0.93 (d, 6H, J = 4.1 Hz), 0.90-0.87 (m, 1H), 0.86 (s, 3H), 0.84 (s, 3H), 0.77 (s, 3H). ^{13}C-NMR (DMSO-d_6, 151 MHz) δ: 178.9, 167.4, 117.6, 115.7, 90.5, 81.0, 74.2, 54.9, 50.4, 44.0, 43.9, 41.8, 40.1, 38.4, 38.1, 37.6, 36.0, 33.7, 33.5, 33.0, 28.2, 27.9, 27.4, 27.3, 23.8, 22.6, 20.8, 18.3, 18.1, 17.2, 16.5, 16.1. IR (neat) v_{max}/cm^{-1} 2934 (m), 2878 (w), 1760 (m), 1733 (w), 1709 (s), 1475 (m), 1285 (w), 1241(s), 1225 (s), 1143 (w), 1021 (w), 985 (w), 948 (s), 820 (m), 728 (m). HRMS-ESI m/z calcd for $C_{38}H_{50}O_7F_2$ $(M+Na)^+$: 679.3417, found 679.3417.

*3,6-Difluoro-2-(((3S,4aR,6aR,6bS,8aS,12aS,12bS,-13S,14aR,14bR)-13-hydroxy-4,4,6a,6b,11,11,14b-heptamethyl-16-oxooctadecahydro-1H,9H-12b,8a-(epoxymethano)-picen-3-yl)oxy)carbonyl)benzoic acid (**52**)*. Compound **52** was obtained from educt 50 (100.0 mg, 0.22 mmol, 1.00 eq) and 3,6-difluorophthalic anhydride (80.6 mg, 0.44 mmol, 2.00 eq) according to general procedure A. Reaction time: 3 days (110 °C). Purification: silica gel chromatography (DCM:MeOH 95:5, silica gel: 23 g). Yield: 33.1 mg (23%). HPLC (method C): t_R = 7.6 min, >99%. ^{1}H-NMR ($CDCl_3$, 600 MHz) δ: 7.19 (td, 1H, J = 8.9, 4.0 Hz), 7.18 (td, 1H, J = 8.9, 3.7 Hz), 4.76 (dd, 1H, J = 11.9, 4.6 Hz), 3.86 (s, 1H), 2.27-2.10 (m, 2H), 1.95-1.90 (m, 3H), 1.79-1.53 (m, 6H), 1.52-1.42 (m, 2H), 1.40-1.31 (m, 3H), 1.27 (s, 3H), 1.19-1.15 (m, 3H), 1.12-1.07 (m, 3H), 1.03 (s, 3H), 1.01-0.95 (m, 1H), 0.93 (d, 6H, J = 4.6 Hz), 0.90-0.87 (m, 1H), 0.86 (s, 3H), 0.85 (s, 3H), 0.78 (s, 3H). ^{13}C-NMR ($CDCl_3$, 151 MHz) δ: 178.8, 90.6, 81.3, 74.2, 54.8, 50.3, 44.0, 43.8, 41.8, 41.6, 38.4, 38.0, 37.5, 36.0, 33.7, 33.4, 33.1, 31.2, 28.2, 27.6, 24.7, 27.3, 23.8, 22.7, 20.7, 18.3, 18.1, 17.2, 16.5, 16.0. IR (neat) v_{max}/cm^{-1} 3511 (w), 2935 (s), 2878 (m), 1762 (m),1719 (s), 1615 (w), 1474 (s), 1280(m), 1243 (m), 1225 (m), 1134 (m), 1022 (w), 948 (s), 820 (m), 728 (m), 673 (w). HRMS-ESI m/z calcd for $C_{38}H_{50}O_7F_2$ $(M+Na)^+$: 679.3417, found 679.3417.

Literature

M. Kvasnica, J. Sarek, E. Klinotova, P. Dzubak, M. Hajduch, Synthesis of Phthalates of Betulinic Acid and Betulin with Cytotoxic Activity. *Bioorg. Med. Chem.* **2005**, *13*, 3447-3454.

2 D. Thibeault, C. Gauthier, J. Legault, J. Bouchard, P. Dufour, A. Pichette, Synthesis and Structure-Activity Relationship Study of Cytotoxiy Germanicane- and Lupane-Type 3β-O-Monodesmosidic Saponins Starting from Betulin. *Bioorg. Med. Chem.* **2007**, *15,* 6144-6157.

3 Y. Yasin, M. Basri, F. Ahmad, A. B. Salleh, Response Surface Methodology as a Tool to Study the Lipase-Catalyzed Synthesis of Betulinic Acid Ester. *J. Chem. Technol. Biotechnol.* **2008**, *83*, 694-698.

4 D. S. H. L. Kim, J. M. Pezzuto, E. Pisha, Synthesis of Betulinic Acid Derivatives with Activity Against Human Melanoma. *Bioorg. Med. Chem. Lett.* **1998**, *8*, 1707-1712.

5 F. Soler, C. Poujade, M. Evers, J.-C. Carry, Y. Hénin, A. Bousseau, T. Huet, R. Pauwels, E. De Clercq, J.-F. Mayaux, J.-B. Le Pecq, N. Dereu, Betulinic Acid Derivatives: A New Class of Specific Inhibitors of Human Immunodeficiency Virus Type 1 Entry. *J. Med. Chem.* **1996**, *39*, 1069-1083.

6 B. P. Pradhan, P. Ghosh, S. Chakraborty, Oxidation of Triterpenoids. Part X. Oxidation of Lupenyl Acetate and Methyl Acetylbetulinate with Selenium Dioxide and Hydrogen Peroxide in Tert-Butanol. *Indian J. Chem.* **1991**, *6*, 549-553.

7 M. Kvasnica, J. Sarek, E. Klinotova, P. Dzubak, M. Hajduch, Synthesis of Phthalates of Betulinic Acid and Betulin with Cytotoxic Activity. *Bioorg. Med. Chem.* **2005**, *13*, 3447-3454.

8 J. L. C. Sousa, C. S. R. Freire, A. J. D. Silvestre, A. M. S. Silva, Recent Developments in the Functionalization of Betulinic Acid and its Natural Analogues: A Route to New Bioactive Compounds. *Molecules* **2019**, *24*, 355.

9 F. Soler, C. Poujade, M. Evers, J.-C. Carry, Y. Hénin, A. Bousseau, T. Huet, R. Pauwels, E. De Clercq, J.-F. Mayaux, J.-B. Le Pecq, N. Dereu, Betulinic Acid Derivatives: A New Class of Specific Inhibitors of Human Immunodeficiency Virus Type 1 Entry. *J. Med. Chem.* **1996**, *39*, 1069-1083.

0 L. F. Tietze, H. Heinzen, P. Moyna, M. Rischer, H. Neunaber, Synthesis of [13C]- and [2H]Betulin for Biological Transformations. *Liebigs Ann. Chem.* **1991***,* 1245-1249.

1 M. N. Gorbunova, G. F. Krainova, I. A. Tolmacheva, V. V. Grishko, New Polymeric Derivatives of Betulin. *Russ. J. Appl. Chem.* **2012**, *85*, 1137-1141.

2 Y. Liang, M. Zhu, T. Xu, W. Ding, M. Chen, Y. Wang, J. Zheng, A Novel Betulinic Acid Analogue: Synthesis, Solubility, Antitumor Activity and Pharmacokinetic Study in Rats. *Molecules* **2023**, *28*, 5715.

3 D. Niewolik, K. Krukiewicz, B. Bednarczyk-Cwynar, P. Ruszkowski, K. Jaszcz, Novel Polymeric Derivatives of Betulin with Anticancer Activity. *RSC Adv.* **2019**, *9*, 20892-20900.

4 S.-M. Kim, I. H. Jeong, M. S. Yim, M. K. Chae, H. N. Kim, D. K. Kim, C. M. Kang, Y. S. Choe, C. Lee, E. K. Ryu, Characterization of Oleanolic Acid Derivatives for Colon Cancer Targeting with Positron Emission Tomography. *J. Drug Target* **2014**, *22,* 191-199.

5 T. Konoike, K. Takahashi, Y. Araki, I. Horibe, Practical Partial Synthesis of Myriceric Acid A, an Endothelin Receptor Antagonist, from Oleanolic Acid. *J. Org. Chem.* **1997**, *62*, 960-966.
